# Analysis of Mortality Among Transgender and Gender Diverse Adults in England

**DOI:** 10.1001/jamanetworkopen.2022.53687

**Published:** 2023-01-30

**Authors:** Sarah S. Jackson, Jalen Brown, Ruth M. Pfeiffer, Duncan Shrewsbury, Stewart O’Callaghan, Alison M. Berner, Shahinaz M. Gadalla, Meredith S. Shiels

**Affiliations:** 1 Infections and Immunoepidemiology Branch, Division of Cancer Epidemiology and Genetics, National Cancer Institute, National Institutes of Health, Rockville, Maryland; 2 Department of Medical Education, Brighton & Sussex Medical School, Brighton, United Kingdom; 3 Live Through This Charity, London, United Kingdom; 4 Barts Cancer Institute, Queen Mary University of London, London, United Kingdom; 5 Gender Identity Clinic, Tavistock and Portman NHS Foundation Trust, London, United Kingdom

## Abstract

**Importance:**

Limited prior research suggests that transgender and gender diverse (TGD) people may have higher mortality rates than cisgender people.

**Objective:**

To estimate overall and cause-specific mortality among TGD persons compared with cisgender persons.

**Design, Setting, and Participants:**

This population-based cohort study used data from general practices in England contributing to the UK’s Clinical Practice Research Datalink GOLD and Aurum databases. Transfeminine (assigned male at birth) and transmasculine (assigned female at birth) individuals were identified using diagnosis codes for gender incongruence, between 1988 and 2019, and were matched to cisgender men and women according to birth year, practice, and practice registration date and linked to the Office of National Statistics death registration. Data analysis was performed from February to June 2022.

**Main Outcomes and Measures:**

Cause-specific mortality counts were calculated for categories of disease as defined by *International Statistical Classification of Diseases and Related Health Problems, Tenth Revision* chapters. Overall and cause-specific mortality rate ratios (MRRs) were estimated using Poisson models, adjusted for index age, index year, race and ethnicity, Index of Multiple Deprivation, smoking status, alcohol use, and body mass index.

**Results:**

A total of 1951 transfeminine (mean [SE] age, 36.90 [0.34] years; 1801 White [92.3%]) and 1364 transmasculine (mean [SE] age, 29.20 [0.36] years; 1235 White [90.4%]) individuals were matched with 68 165 cisgender men (mean [SE] age, 33.60 [0.05] years; 59 136 White [86.8%]) and 68 004 cisgender women (mean [SE] age, 33.50 [0.05] years; 57 762 White [84.9%]). The mortality rate was 528.11 deaths per 100 000 person-years (102 deaths) for transfeminine persons, 325.86 deaths per 100 000 person-years (34 deaths) for transmasculine persons, 315.32 deaths per 100 000 person-years (1951 deaths) for cisgender men, and 260.61 deaths per 100 000 person-years (1608 deaths) for cisgender women. Transfeminine persons had a higher overall mortality risk compared with cisgender men (MRR, 1.34; 95% CI, 1.06-1.68) and cisgender women (MRR, 1.60; 95% CI, 1.27-2.01). For transmasculine persons, the overall MMR was 1.43 (95% CI, 0.87-2.33) compared with cisgender men and was 1.75 (95% CI, 1.08-2.83) compared with cisgender women. Transfeminine individuals had lower cancer mortality than cisgender women (MRR, 0.52; 95% CI, 0.32-0.83) but an increased risk of external causes of death (MRR, 1.92; 95% CI, 1.05-3.50). Transmasculine persons had higher mortality from external causes of death than cisgender women (MRR, 2.77; 95% CI, 1.15-6.65). Compared with cisgender men, neither transfeminine nor transmasculine adults had a significantly increased risk of deaths due to external causes.

**Conclusions and Relevance:**

In this cohort study of primary care data, TGD persons had elevated mortality rates compared with cisgender persons, particularly for deaths due to external causes. Further research is needed to examine how minority stress may be contributing to deaths among TGD individuals to reduce mortality.

## Key Points

**Question:**

Do transgender and gender diverse (TGD) individuals have increased overall and cause-specific morality compared with cisgender people?

**Findings:**

In this cohort study of 139 484 individuals, TGD people had elevated overall mortality compared with cisgender people, specifically deaths from external causes (suicides, homicides, and accidental poisonings), endocrine disorders, and other ill-defined and unspecified causes. Transfeminine individuals had a decreased cancer mortality risk compared with cisgender women but the same risk as cisgender men, whereas transmasculine individuals had the same cancer mortality risk as cisgender people.

**Meaning:**

These findings highlight the need to develop interventions to prevent suicide, homicide, and accidental poisonings to reduce mortality for TGD individuals.

## Teaser

This cohort study estimates overall and cause-specific mortality among transgender and gender diverse persons compared with cisgender persons in the UK.

## Introduction

Transgender and gender diverse (TGD) individuals have gender identities that differ from their assigned birth sex. In the UK, approximately 200 000 to 500 000 persons older than 16 years (1% of the population) consider themselves to be TGD.^1^ Mortality among TGD persons may be higher than that of cisgender persons (individuals whose assigned birth sex matches their gender identity) because of increased risk of external causes of death and deaths due to illness. The murder of TGD people has been increasing globally, particularly for transgender women, who account for 96% of homicides among TGD people.^2^ In the UK, transphobic hate crimes reported to the police have quadrupled over the past 6 years.^3^ Research from the US^4,5^ suggests that TGD people are likely to experience violence and that self-harm is common among these individuals, with 41% reporting at least 1 suicide attempt in their lifetimes.

The minority stress model posits that chronic stress due to repeated exposure to violence, discrimination, and economic and social marginalization results in greater vulnerability to poor health outcomes and mortality among TGD individuals globally.^6^ The prevalence of alcohol abuse and tobacco use has been reported to be higher among TGD individuals than cisgender individuals.^7,8^ The global HIV prevalence among transgender women is 19%, nearly 50 times that of cisgender people.^9^ Cancer mortality for specific sites has also been reported to be higher among transgender individuals than cisgender individuals.^10,11^ TGD persons may also be at increased risk of mortality because of the long-term use of gender-affirming hormone therapy. Limited evidence^12,13^ suggests that estrogen use may increase the risk of myocardial infarction and ischemic stroke in transgender women. Research^14,15^ indicates that transgender men have a 2-fold and 4-fold increased rate of myocardial infarction compared with cisgender men and cisgender women, respectively, likely due to testosterone therapy and chronic stress resulting from discrimination and minoritized status.

Previous analyses^11,16,17^ have focused on TGD people who use gender-affirming hormone therapy, thereby excluding those who do not use hormones but may have poorer health outcomes associated with marginalized group status. Many prior analyses were either based on small sample size^18,19^ or did not adjust for important risk factors for mortality (eg, smoking, body mass index, or alcohol use).^11,20,21^ We sought to examine overall and cause-specific mortality in a cohort of TGD individuals compared with a matched cohort of cisgender people.

## Methods

This cohort study used 2 primary care databases, Clinical Practice Research Datalink (CPRD) GOLD and CPRD Aurum. CPRD GOLD was established in 1987 and contains the primary care data from approximately 9% of the UK, whereas CPRD Aurum was established in 1995 and covers only English practices (approximately 13% of England).^22,23^ Both databases are representative of the general population in terms of age and sex.^22,23^ In CPRD, patient demographics, clinical diagnoses, symptoms, medications, and specialist referrals are recorded by general practitioners using either Read (GOLD) or SNOMED (Aurum) codes.^22,23^

This study is based on data from the February 2020 CPRD GOLD and Aurum database releases (obtained under license from the UK Medicines and Healthcare products Regulatory Agency; the data are provided by patients and collected by the National Health Service as part of their care and support) and the Office of National Statistics (ONS) and Hospital Episode Statistics (HES) releases (Linkage Set 18; reused with the permission of The Health & Social Care Information Centre). The study was approved by the Independent Scientific Advisory Committee of the CPRD. Informed consent of individual patients was not required because anonymized information was obtained from medical records. This study follows Strengthening the Reporting of Observational Studies in Epidemiology (STROBE) reporting guideline for cohort studies.^24^

We used diagnosis codes for gender incongruence (previously coded as gender dysphoria or gender identity disorder) to identify 7151 TGD patients in CPRD who were aged 18 years or older (eTables 1, 2, 3, and 4 in Supplement 1). The date of the first qualifying gender incongruence term was set to the later of the index date or date of 18th birthday. Identified TGD individuals were then individually matched to 20 cisgender men and 20 cisgender women who were alive on the TGD person’s index date on the following variables: year of birth (within 1 year), practice, and date of practice registration (within 5 years). Inclusion criteria for both groups must have been recorded after the date CPRD identified the practice recording to be up to standard.^23^ All individuals with diagnostic codes for variations of sex characteristics (formerly differences of sex development) were excluded.

In CPRD, sex assigned at birth and gender identity are not collected separately. For TGD individuals, sex assigned at birth was determined from documentation of gender-affirming therapies and surgical procedures, sex-specific procedures listed in the primary care database, and the linked HES files (eAppendix and eTables 5, 6, 7, and 8 in Supplement 1). We classified TGD individuals as transfeminine (assigned male at birth), transmasculine (assigned female at birth), or TGD, unknown sex assigned at birth, with the understanding that some individuals, particularly those with nonbinary identities, may not identify with these classifications.

Only patients from consenting English practices were eligible to be linked to several registries including the ONS death registration and the HES files. Patients could be represented in the death registry more than once if their practice switched from GOLD to Aurum (all patient data were backfilled from GOLD to Aurum if this occurred). CPRD deduplicated the linked ONS and HES files before analysis to prevent double counting these patients. The death registry records all deaths, including underlying cause of and date of death, occurring in England and covers the period January 2, 1998, to May 31, 2019. Causes of deaths were coded using the *International Statistical Classification of Diseases and Related Health Problems, Tenth Revision*. Fifty-two percent of the individuals from CPRD (33% from GOLD and 79% from Aurum) were eligible to be linked to the ONS death registry. After data cleaning and exclusions, the final analytic data set consisted of 139 486 individuals, including 3317 TGD and 136 169 cisgender individuals (eAppendix and eFigure in Supplement 1).

### Statistical Analysis

Data analysis was performed from February to June 2022. We calculated the distribution of cohort characteristics by gender identity as frequencies and percentages, means and SDs, and medians and IQRs. Absolute numbers and proportions for counts of 5 or fewer were suppressed because of privacy concerns. We used Poisson regression models to estimate the mortality rate ratios (MRRs) and 95% CIs for overall and cause-specific mortality in TGD individuals (transfeminine, transmasculine, or TGD unknown sex assigned at birth) compared with cisgender men and cisgender women separately. All models were adjusted for continuous index age, continuous index year, race and ethnicity (White, Black, Asian, or another or unknown race and ethnicity), Index of Multiple Deprivation (socioeconomic status measure, in quintiles), smoking status (current, former, or never), alcohol use (current, former, never), body mass index, calculated as weight in kilograms divided by height in meters squared (underweight or healthy weight [<18.5-24.9], overweight [25.0-29.9], or obese [≥30.0]), including the log of person-years as an offset term and a random intercept for practice to account for the correlation between patients from the same practice. Race and ethnicity were identified in the HES database and were assessed in this study because mortality rates vary by racial and ethnic group. Because death dates are also recorded in the CPRD primary care database and have been confirmed to be complete and accurate,^25^ person-time was calculated as the time from the index date to the date of death (recorded in CPRD or the death registry) or end of the death registry coverage period (May 31, 2019). Cause-specific mortality counts were calculated for categories of disease as defined by *International Statistical Classification of Diseases and Related Health Problems, Tenth Revision* chapters (eg, infection, cancer, and nonnatural causes of death), and MRRs were calculated for categories with deaths among TGD persons. Mortality risks were estimated for the 10 chapters that had more than 0 deaths among TGD people. Where the sample size was sufficient, we further divided categories of diseases into the individual cause of death categories suicide or homicide, accidental poisonings, gastrointestinal and lung cancers (the 2 most common cancers among TGD people), endocrine disorders, and other ill-defined and unspecified causes of mortality. Because we identified TGD persons from diagnosis codes related to gender incongruence but were not required to have a record of gender-affirming care (hormones or surgery), we could not determine the sex assigned at birth for 33% of TGD individuals. Therefore, we used multiple imputation with chained equations to impute missing values for sex assigned at birth and other variables with missing values (eAppendix in Supplement 1).^26^ The imputed results are presented as our main analysis. The results without imputation for sex assigned at birth and other missing data are presented in eTables 9 and 10 in Supplement 1.

We also conducted a sensitivity analysis restricted to individuals who were alive and enrolled in a CPRD practice as of January 2, 1998 (the start of ONS death registry coverage). In this analysis, person-time was calculated from the later of the index date or January 2, 1998, to the date of death or May 31, 2019 (end of ONS death registry coverage), using multiple imputation. All analyses were conducted using SAS statistical software version 9.4 (SAS Institute).

## Results

After imputing missing sex assigned at birth, the cohort consisted of 1951 transfeminine (1801 White [92.3%]) and 1364 transmasculine individuals (1235 White [90.4%]) matched to 68 165 cisgender men (59 136 White [86.8%]) and 68 004 cisgender women (57 762 White [84.9%]) (Table 1). Transmasculine adults were younger (mean [SE] age at index date, 29.20 [0.36] years) than transfeminine individuals (mean [SE], 36.90 [0.34] years), cisgender men (mean [SE], 33.60 [0.05] years), and cisgender women (mean [SE], 33.50 [0.05] years). Transmasculine adults were less likely to be current alcohol users and more likely to be obese than other gender identities. Transfeminine individuals were more likely to be White and current smokers than cisgender people.

**Table 1.** Characteristics of Transgender and Gender Diverse Individuals and Cisgender Individuals in the UK’s Clinical Practice Research Datalink, 1988 to 2019, Using Multiple Imputation^a^

Characteristic	Patients, No. (%)			
	Transfeminine individuals (n = 1951)	Transmasculine individuals (n = 1364)	Cisgender men (n = 68 165)	Cisgender women (n = 68 004)
Age, mean (SE), y	36.9 (0.34)	29.2 (0.36)	33.6 (0.05)	33.5 (0.05)
Body mass index^b^				
Underweight or normal (<18.5-24.9)	948 (48.6)	664 (48.7)	31 110 (45.6)	34 809 (51.2)
Overweight (25.0-29.9)	569 (29.2)	327 (23.9)	23 152 (34.0)	17 333 (25.5)
Obese (≥30.0)	434 (22.3)	374 (27.4)	13 903 (20.4)	15 862 (22.3)
Smoking status				
Never	656 (33.7)	511 (31.4)	23 254 (34.1)	18 517 (27.2)
Former	654 (33.5)	426 (37.4)	26 875 (39.4)	29 415 (43.3)
Current	640 (32.8)	657 (31.2)	18 036 (26.5)	20 072 (29.5)
Alcohol use				
Never	143 (7.3)	141 (10.3)	5077 (7.4)	6714 (9.9)
Former	113 (5.8)	91 (6.7)	2631 (3.9)	3526 (5.2)
Current	1696 (86.9)	1133 (83.0)	60 457 (88.7)	57 764 (84.9)
Race and ethnicity^c^				
Asian, unknown, or another race	123 (6.3)	95 (7.0)	6471 (9.5)	7218 (10.6)
Black	28 (1.4)	35 (2.6)	2558 (3.8)	3024 (4.4)
White	1801 (92.3)	1235 (90.4)	59 136 (86.8)	57 762 (84.9)
Index of Multiple Deprivation, quintile^d^				
First	247 (12.6)	162 (11.9)	8346 (12.2)	8310 (12.2)
Second	3289 (16.8)	202 (14.8)	10 723 (15.7)	10 722 (15.8)
Third	374 (19.2)	241 (17.7)	12 680 (18.6)	12 673 (18.6)
Fourth	477 (24.5)	378 (27.7)	17 605 (25.8)	17 578 (25.8)
Fifth	526 (27.0)	382 (28.0)	18 811 (27.6)	18721 (27.5)
Person-time, y				
Median (IQR)	9.2 (12.4)	5.2 (10.7)	7.5 (12.5)	7.5 (12.5)
Total	19 314	10 434	618 733	617 015
Died	102 (5.2)	34 (2.5)	1951 (2.9)	1608 (2.4)
Mortality rate, deaths per 100 000 persons	528.11	325.86	315.32	260.61
Cause of death				
Certain infectious and parasitic diseases	≤5^e^	0	19 (1.0)	29 (1.9)
Codes for special purposes	≤5^e^	0	12 (0.7)	≤5^e^
Congenital malformations, deformations, and chromosomal abnormalities	0	0	7 (0.4)	≤5^e^
Diseases of the blood and blood-forming organs and certain disorders involving the immune mechanism	0	0	6 (0.3)	6 (0.4)
Diseases of the circulatory system	25 (26.5)	7 (19.7)	506 (28.0)	294 (19.3)
Diseases of the digestive system	9 (9.5)	≤5^e^	152 (8.3)	111 (7.3)
Diseases of the eye and adnexa	0	0	0	≤5^e^
Diseases of the genitourinary system	0	0	18 (1.0)	17 (1.1)
Diseases of the musculoskeletal system and connective tissue	0	0	7 (0.4)	17 (1.1)
Diseases of the nervous system	≤5^e^	0	84 (4.6)	86 (5.7)
Diseases of the respiratory system	10 (10.4)	≤5^e^	203 (11.0)	156 (10.3)
Diseases of the skin and subcutaneous tissue	0	0	6 (0.3)	≤5^e^
Endocrine, nutritional, and metabolic diseases	≤5^e^	≤5^e^	35 (1.9)	19 (1.3)
External causes of morbidity and mortality	13 (13.9)	8 (22.7)	169 (9.2)	98 (6.4)
Mental and behavioral disorders	≤5^e^	≤5^e^	67 (3.6)	83 (5.5)
Neoplasms	20 (20.4)	8 (25.3)	530 (28.8)	580 (38.2)
Pregnancy, childbirth, and the puerperium	0	0	0	≤5^e^
Symptoms, signs and abnormal clinical and laboratory findings, not elsewhere classified	≤5^e^	≤5^e^	19 (1.0)	9 (0.6)

^a^ Sex assigned at birth for transgender individuals where it was previously unknown was imputed using multiple imputation with chained equations. Body mass index, smoking status, and alcohol use were imputed for the entire cohort.

^b^ Body mass index is calculated as weight in kilograms divided by height in meters squared.

^c^ Asian was defined as Bangladeshi, Chinese, Indian, Pakistani, or other Asian; Black was defined as Black African, Black Caribbean, or Black other; and other was defined as multiracial or any other race or ethnicity.

^d^ Refers to Practice Level Index of Multiple Deprivation.

^e^ Clinical Practice Research Datalink requires suppression of counts ≤5. As such, no proportions are reported for these cells.

During follow-up, the mortality rates were 528.11 deaths per 100 000 person-years (102 deaths) for transfeminine persons and 325.86 deaths per 100 000 person-years (34 deaths) for transmasculine persons. In comparison, the mortality rates were 315.32 deaths per 100 000 person-years (1951 deaths) for cisgender men and 260.61 deaths per 100 000 person-years (1608 deaths) for cisgender women (Table 1). As shown in Table 2, TGD people had an overall increased risk of mortality compared with cisgender people. Compared with cisgender men, there was an increased risk of overall mortality for transfeminine (MRR, 1.34; 95% CI, 1.06-1.68) and transmasculine (MRR, 1.43; 95% CI, 0.87-2.33) adults. Compared with cisgender women, there was an increased risk of overall mortality among transfeminine (MRR, 1.60; 95% CI, 1.27-2.01) and transmasculine (MRR, 1.75; 95% CI, 1.08-2.83) adults. In the nonimputed analysis (eTable 10 in Supplement 1), TGD individuals with unknown sex assigned at birth had the highest risk of death compared with cisgender men (MRR, 1.71; 95% CI, 1.31-2.23) and cisgender women (MRR, 2.11; 95% CI, 1.61-2.78). The results were not materially different when the analysis was restricted to those with follow-up after January 2, 1998 (eTable 11 in Supplement 1).

**Table 2.** Overall and Cause-Specific MMRs for Transgender and Gender Diverse Individuals Compared with Cisgender Individuals in the UK’s Clinical Practice Research Datalink^a^

Cause of death	Transfeminine individuals			Transmasculine individuals		
	No. who died	MRR (95% CI)		No. who died	MRR (95% CI)	
		Compared with cisgender men	Compared with cisgender women		Compared with cisgender men	Compared with cisgender women
Overall	102	1.34 (1.06-1.68)	1.60 (1.27-2.01)	34	1.43 (0.87-2.33)	1.75 (1.08-2.83)
Certain infectious and parasitic diseases	≤5^b^	1.82 (0.41-8.01)	1.02 (0.24-4.43)	0	NA	NA
Diseases of the circulatory system	25	1.03 (0.66-1.59)	1.51 (0.94-2.42)	7	0.83 (0.28-2.46)	1.24 (0.41-3.77)
Diseases of the digestive system	9	1.15 (0.50-2.66)	1.14 (0.50-2.63)	≤5^b^	1.40 (0.16-12.07)	1.45 (0.16-13.11)
Diseases of the nervous system	≤5^b^	0.78 (0.21-2.83)	0.54 (0.14-2.07)	0	NA	NA
Diseases of the respiratory system	10	0.94 (0.47-1.92)	0.95 (0.48-1.89)	≤5^b^	0.89 (0.14-5.74)	0.98 (0.16-5.90)
Endocrine, nutritional, and metabolic diseases	≤5^b^	1.81 (0.59-5.53)	2.84 (0.87-9.24)	≤5^b^	1.91 (0.29-12.60)	3.11 (0.46-20.90)
External causes of mortality	13	1.39 (0.77-2.49)	1.92 (1.05-3.50)	8	1.84 (0.81-4.17)	2.77 (1.15-6.65)
Mental and behavioral disorders	≤5^b^	0.96 (0.34-2.68)	0.87 (0.32-2.42)	≤5^b^	0.93 (0.12-7.02)	0.72 (0.10-5.31)
Neoplasms	20	0.78 (0.50-1.22)	0.52 (0.32-0.83)	8	1.06 (0.50-2.27)	0.71 (0.32-1.56)
Symptoms, signs, and abnormal clinical and laboratory findings, not elsewhere classified	≤5^b^	4.87 (1.71-13.89)	3.49 (0.47-25.79)	≤5^b^	9.27 (2.93-29.30)	6.83 (0.88-52.74)

Abbreviations: MRR, mortality rate ratio; NA, not applicable.

^a^ Missing sex assigned at birth and covariate data were imputed using multiple imputation. Models were estimated using Poisson regression adjusted for index age, index year, race and ethnicity (White, Black, Asian, or another or unknown race or ethnicity), Index of Multiple Deprivation (quintiles), smoking status (current, former, or never), alcohol use (current, former, or never), body mass index (weight in kilograms divided by height in meters squared: underweight or healthy weight [<18.5-24.9], overweight [25.0-29.9], or obese [≥30.0]), and practice.

^b^ Clinical Practice Research Datalink requires suppression of counts ≤5.

Cause-specific mortality is presented in Table 2. The risk of cancer death was decreased for transfeminine adults compared with cisgender women (MRR, 0.52; 95% CI, 0.32-0.83) but not when compared with cisgender men or for transmasculine adults compared with either cisgender group. Compared with cisgender women, there was an increased risk of death from external causes for transfeminine (MRR, 1.92; 95% CI, 1.05-3.50) and transmasculine (MRR, 2.77; 95% CI, 1.15-6.65) adults. Compared with cisgender men, neither transfeminine adults nor transmasculine adults had a significantly increased risk of deaths due to external causes. Transfeminine adults had a significantly increased of mortality risk from diseases with symptoms, signs, and abnormal clinical and laboratory findings, not elsewhere classified, compared with cisgender men (MRR, 4.87; 95% CI, 1.71-13.89) but not cisgender women (MRR, 3.49; 95% CI, 0.47-25.79). The risk of mortality in this classification was elevated for transmasculine adults compared with cisgender men (MRR, 9.27; 95% CI, 2.93-29.30) but was not significant when compared with cisgender women (MRR, 6.83; 95% CI, 0.88-52.74). Results from the nonimputed analysis were similar to the main findings (eTable 10 in Supplement 1), with the exception that TGD persons with unknown sex assigned at birth had increased mortality due to external causes compared with cisgender men (MRR, 1.82; 95% CI, 1.31-2.23) and cisgender women (MRR, 2.11; 95% CI, 1.61-2.78). These results did not differ when we restricted follow-up time (eTable 11 in Supplement 1).

We investigated deaths due to external causes; neoplasms; endocrine, nutritional, and metabolic diseases; and symptoms, signs, and abnormal clinical and laboratory findings, not elsewhere classified, by comparing all TGD individuals combined with cisgender men and cisgender women separately (Table 3). Compared with cisgender men, TGD people were 3 times as likely to die from suicide or homicide (MRR, 3.34; 95% CI, 1.70-6.54) and more than twice as likely to die from accidental poisonings (MRR, 2.28; 95% CI, 1.04-5.02). Compared with cisgender women, TGD people were more than 5 times as likely to die from suicide or homicide (MRR, 5.62; 95% CI, 2.65-11.91) and from accidental poisonings (MRR, 5.20; 95% CI, 2.22-12.18). We examined the most common cancer deaths occurring among TGD people: gastrointestinal (consisting of esophagus, stomach, colorectal, and pancreas cancers) and lung cancer. We found no difference in mortality from these 2 cancers between TGD people and cisgender people. In contrast, there were 51 prostate cancer deaths in cisgender men and none among transfeminine adults, and there were 116 breast cancer deaths among cisgender women but none among transmasculine adults. TGD individuals combined had increased mortality from endocrine diseases compared with cisgender women (MRR, 2.95; 95% CI, 1.08-8.07), but not cisgender men (MRR, 1.80; 95% CI, 0.69-4.66). TGD people were more likely to have their deaths classified as symptoms, signs, and abnormal clinical and laboratory findings, not elsewhere classified than cisgender men (MRR, 5.27; 95% CI, 1.95-14.26) and cisgender women (MRR, 18.63; 95% CI, 5.39-64.37). These results did not differ when we excluded those with missing data (eTable 12 in Supplement 1) or restricted follow-up time (eTable 13 in Supplement 1).

**Table 3.** MRRs for Select Causes Among Transgender and Gender Diverse Individuals Compared With Cisgender Individuals in the UK’s Clinical Practice Research Datalink^a^

Cause of death	All transgender and gender diverse individuals		
	No. who died	MRR (95% CI)	
		Compared with cisgender men	Compared with cisgender women
External causes of mortality			
Suicide or homicide	10	3.34 (1.70-6.54)	5.62 (2.65-11.91)
Accidental poisoning	7	2.28 (1.04-5.02)	5.20 (2.22-12.18)
Neoplasms			
Gastrointestinal	9	1.15 (0.50-2.66)	1.14 (0.50-2.63)
Lung	14	1.28 (0.65-2.52)	1.22 (0.62-2.41)
Endocrine, nutritional, and metabolic diseases	≤5^b^	1.80 (0.69-4.66)	2.95 (1.08-8.07)
Symptoms, signs and abnormal clinical and laboratory findings, not elsewhere classified; other ill-defined and unspecified causes of mortality	≤5^b^	5.27 (1.95-14.26)	18.63 (5.39-64.37)

Abbreviation: MRR, mortality rate ratio.

^a^ Missing covariate data were imputed using multiple imputation. Models were estimated using Poisson regression adjusted for continuous index age, continuous index year, race and ethnicity (White, Black, Asian, or another or unknown race or ethnicity), Index of Multiple Deprivation (quintiles), smoking status (current, former, never), alcohol use (current, former, never), body mass index (weight in kilograms divided by height in meters squared: underweight or healthy weight [<18.5-24.9], overweight [25.0-29.9], or obese [≥30.0]), and practice.

^b^ Clinical Practice Research Datalink requires suppression of counts ≤5.

## Discussion

This cohort study found that TGD individuals have an increased risk of overall mortality, ranging from 34% to 75%, compared with cisgender people. We found an increased mortality due to external causes, with nearly 2-fold increased risk of death among transfeminine adults and almost a 3-fold risk of death among transmasculine adults compared with cisgender women. Research from the Netherlands^11^ found the risk of mortality for transgender women was 1.6 times that of cisgender men and 2.4 times that of cisgender women. In that analysis, transgender men had 1.6 times the mortality risk of cisgender women but a nonsignificant increased risk of death that was 1.1 times that of cisgender men.^11^ In our analysis, we found TGD persons with unknown sex assigned at birth had mortality rates 1.71 to 2.11 times higher than those for cisgender men and women, respectively. Similarly, Hughes et al^27^ found that individuals for whom they were unable to determine sex assigned at birth had the highest mortality rates. This group, which consists of nonbinary persons who may not seek gender-affirming care, those who access hormone therapy outside the medical system, or those with limited access to medical care because of fears of discrimination and other barriers to health care, may experience higher levels of minority stress than TGD persons with a record of gender-affirming care.^7^

Previous research^11,16,17,18,20,21,28^ has found mortality from external causes is 2 to 19 times higher in TGD persons than cisgender persons. When we compared all TGD people combined with cisgender people, we saw 3 to 5 times greater increased mortality from suicide or homicide and 2 to 5 times increased mortality from accidental poisonings. However, when we separately compared transfeminine individuals and transmasculine individuals with cisgender persons, we found an increased risk of external causes of death compared with cisgender women only. Historically, across all age groups, cisgender women have had lower mortality rates due to external causes of death compared with cisgender men.^29^ De Blok et al^11^ found a 3- to 7-fold increased risk of suicide among transgender women compared with cisgender people, but no significant increase among transgender men. Forty-one percent of TGD respondents in the US National Transgender Discrimination Survey reported attempting suicide, a proportion much greater than the 5% of US adults and the 10% to 20% of lesbian, gay, and bisexual adults who reported ever attempting suicide.^4^ Factors associated with suicide attempts included experiencing minority stress related to comorbid health conditions, alienation from family, and experiencing discrimination or marginalization at school or work or when accessing health care.^4^

The rate of deaths coded as symptoms, signs, and abnormal clinical and laboratory findings, not elsewhere classified, were 5 to 19 times higher among TGD individuals compared with cisgender persons. These codes are used when the physician completing the death certificate has insufficient knowledge of the disease that caused the death. These codes are often temporary, because an autopsy can determine cause of death to update the death certificate.^30^ In the present study, all these deaths occurred well before the end of the death registry. Previous research^30,31^ has shown that these codes are more commonly used among minoritized ethnic groups and those from marginalized populations. However, given the small number of events in our study, it is difficult to make inferences about these results.

We did not find increased deaths from cardiovascular infectious diseases among TGD persons in our analysis, contrary to previous research.^11,32^ Our TGD cohort is young, and cardiovascular deaths in these age groups are rare. Furthermore, we included TGD persons without documentation of gender-affirming hormone therapy, so cardiovascular risk may be lower in our cohort than if we had restricted to those taking therapy. Because we used a primary care database of people receiving medical care, the individuals living with HIV in our study may be more likely to be receiving treatment than individuals sampled in other studies.^11,16,17^ Furthermore, the median index year in our study was 2011, when the use of highly active antiretroviral therapy was widespread, resulting in greatly reduced HIV-related mortality.^33^ Indeed, in previous studies,^11^ HIV-related deaths decreased among TGD people over time owing to improved HIV treatment. However, mortality from endocrine disorders was more common among TGD individuals compared with cisgender women, which, to our knowledge, has not been reported before.^11,17^ Further research is needed to understand this increased risk.

We found that TGD persons had no increased risk of cancer death, with transfeminine adults having half the mortality risk of cisgender men. When we examined the most common cancer-specific deaths among TGD people (gastrointestinal and lung cancers), we did not find differences in mortality between TGD individuals and cisgender individuals. Lower rates of cancer-specific mortality among transfeminine people compared with cisgender men may be due to deaths from prostate cancer. There were 51 prostate cancer deaths in cisgender men, and none among transfeminine adults, who have a substantially lower risk of prostate cancer owing to the use of antiandrogen and estrogen hormone therapy.^34^ Similarly, there were 116 breast cancer deaths among cisgender women but none among transmasculine adults, for whom risk is lower because of gender-affirming chest surgery.^35^ However, as noted already, our cohort is young, and more longitudinal data are needed to understand how long-term hormone use may affect cancer risk at older ages.

### Limitations

This study has limitations. CPRD does not collect gender identity from self-report, which is the criterion standard for ascertaining TGD status.^36^ Instead, we used diagnosis codes for gender incongruence to identify TGD patients, which may have missed individuals and underestimated mortality among TGD persons. We used multiple imputation to fill in missing sex assigned at birth for approximately 1000 TGD individuals, which is an imperfect solution and highlights the need for the collection of self-reported gender identity in medical systems. Hormone therapy prescribed by the gender identity clinics was not consistently updated in the patient’s chart. Missing information on hormone therapy prevented us from elucidated sex assigned at birth for many patients and prevented us from examining associations between hormone use and some causes of death (eg, endocrine disorders). We were not able to able to analyze individuals of Asian ethnicity or another race separately because of a limited sample size. In addition, although this is one of the largest mortality studies of TGD persons, the number of deaths was small, thus limiting its statistical power.

## Conclusions

To our knowledge, this cohort study is the first report on mortality among TGD persons in England. We found that transfeminine and transmasculine individuals had higher mortality rates than cisgender individuals. Furthermore, we found that those without a record of gender-affirming care and nonbinary individuals may be at highest risk of mortality, underscoring the need for customized care for this population. Our results highlight the need to understand the factors leading to minority stress and to develop interventions for suicide, homicide, and accidental poisonings to reduce mortality for TGD individuals. Future work should follow this cohort as they age to estimate the incidence of conditions like cardiovascular disease and cancer.

## Notes

Supplement 1. **eAppendix.** Supplemental Methods **eTable 1.** Read Codes and Terms to Identify Transgender and Gender Diverse Individuals in CPRD GOLD **eTable 2.** SNOMED Codes and Terms to Identify Transgender and Gender Diverse Individuals in CPRD Aurum **eTable 3.** Read Codes and Terms to Exclude Individuals With Variations of Sex Characteristics in CPRD GOLD **eTable 4.** SNOMED Codes and Terms to Exclude Individuals With Variations of Sex Characteristics in CPRD Aurum **eTable 5.** Medications Extracted From the Medical Record Used to Identify Sex Assigned at Birth Among Transgender and Gender Diverse Participants in CPRD Aurum and GOLD **eTable 6.** Read Codes and Terms to Identify Sex Assigned at Birth for Transgender and Gender Diverse Individuals in CPRD GOLD **eTable 7.** SNOMED Codes and Terms to Identify Sex Assigned at Birth for Transgender and Gender Diverse Individuals in CPRD Aurum **eTable 8.** Sex-Specific Procedures Extracted From the Hospital Episode Statistics Admitted Patient Care and Outpatient Care Files Used to Identify Sex Assigned at Birth Among Transgender and Gender Diverse Individuals **eTable 9.** Characteristics of Transgender and Gender Diverse Individuals and Cisgender Individuals in the United Kingdom’s Clinical Practice Research Datalink 1988–2019 (Without Imputation) **eTable 10.** Overall and Cause-Specific Mortality Rate Ratios for Transgender and Gender Diverse Individuals Compared to Cisgender Individuals in the United Kingdom’s Clinical Practice Research Datalink (Without Imputation) **eTable 11.** Overall and Cause-Specific Mortality Rate Ratios for Transgender and Gender Diverse Individuals Compared to Cisgender Individuals in the United Kingdom’s Clinical Practice Research Datalink (Only Patients Who Could Have Died During or After 1998) **eTable 12.** Mortality Rate Ratios for Deaths Due to Select Causes of Death Among Transgender and Gender Diverse Individuals Compared to Cisgender Individuals in the United Kingdom’s Clinical Practice Research Datalink (Without Imputation) **eTable 13.** Mortality Rate Ratios for Deaths Due to Select Causes Among Transgender and Gender Diverse Individuals Compared to Cisgender Individuals in the United Kingdom’s Clinical Practice Research Datalink (Only Patients Who Could Have Died During or After 1998) **eFigure.** Flow Chart of Final Analysis Cohort Combining CPRD GOLD and CPRD Aurum

Supplement 2.
Data Sharing Statement
