# Supplementary material for: Analysis of Mortality Among Transgender and Gender Diverse Adults in England
Source: JAMA Netw Open. 2023 Jan 30;6(1):e2253687. doi: 10.1001/jamanetworkopen.2022.53687 (PMC9887492; doi:10.1001/jamanetworkopen.2022.53687)
Supplement: Supplement 1. — eAppendix. Supplemental Methods eTable 1. Read Codes and Terms to Identify Transgender and Gender Diverse Individuals in CPRD GOLD eTable 2. SNOMED Codes and Terms to Identify Transgender and Gender Diverse Individuals in CPRD Aurum eTable 3. Read Codes and Terms to Exclude Individuals With Variations of Sex Characteristics in CPRD GOLD eTable 4. SNOMED Codes and Terms to Exclude Individuals With Variations of Sex Characteristics in CPRD Aurum eTable 5. Medications Extracted From the Medical Record Used to Identify Sex Assigned at Birth Among Transgender and Gender Diverse Participants in CPRD Aurum and GOLD eTable 6. Read Codes and Terms to Identify Sex Assigned at Birth for Transgender and Gender Diverse Individuals in CPRD GOLD eTable 7. SNOMED Codes and Terms to Identify Sex Assigned at Birth for Transgender and Gender Diverse Individuals in CPRD Aurum eTable 8. Sex-Specific Procedures Extracted From the Hospital Episode Statistics Admitted Patient Care and Outpatient Care Files Used to Identify Sex Assigned at Birth Among Transgender and Gender Diverse Individuals eTable 9. Characteristics of Transgender and Gender Diverse Individuals and Cisgender Individuals in the United Kingdom’s Clinical Practice Research Datalink 1988–2019 (Without Imputation) eTable 10. Overall and Cause-Specific Mortality Rate Ratios for Transgender and Gender Diverse Individuals Compared to Cisgender Individuals in the United Kingdom’s Clinical Practice Research Datalink (Without Imputation) eTable 11. Overall and Cause-Specific Mortality Rate Ratios for Transgender and Gender Diverse Individuals Compared to Cisgender Individuals in the United Kingdom’s Clinical Practice Research Datalink (Only Patients Who Could Have Died During or After 1998) eTable 12. Mortality Rate Ratios for Deaths Due to Select Causes of Death Among Transgender and Gender Diverse Individuals Compared to Cisgender Individuals in the United Kingdom’s Clinical Practice Research Datalink (Without Imputation) eTable [file jamanetwopen-e2253687-s001.pdf]

Supplemental Online Content

Jackson SS, Brown J, Pfeiffer RM, et al. Analysis of mortality among transgender and gender diverse adults in England. *JAMA Netw Open*. 2023;6(1):e2253687. doi:10.1001/jamanetworkopen.2022.53687

eAppendix. Supplemental Methods

eTable 1. Read Codes and Terms to Identify Transgender and Gender Diverse Individuals in CPRD GOLD

eTable 2. SNOMED Codes and Terms to Identify Transgender and Gender Diverse Individuals in CPRD Aurum

eTable 3. Read Codes and Terms to Exclude Individuals With Variations of Sex Characteristics in CPRD GOLD

eTable 4. SNOMED Codes and Terms to Exclude Individuals With Variations of Sex Characteristics in CPRD Aurum

eTable 5. Medications Extracted From the Medical Record Used to Identify Sex Assigned at Birth Among Transgender and Gender Diverse Participants in CPRD Aurum and GOLD

eTable 6. Read Codes and Terms to Identify Sex Assigned at Birth for Transgender and Gender Diverse Individuals in CPRD GOLD

eTable 7. SNOMED Codes and Terms to Identify Sex Assigned at Birth for Transgender and Gender Diverse Individuals in CPRD Aurum

eTable 8. Sex-Specific Procedures Extracted From the Hospital Episode Statistics Admitted Patient Care and Outpatient Care Files Used to Identify Sex Assigned at Birth Among Transgender and Gender Diverse Individuals

eTable 9. Characteristics of Transgender and Gender Diverse Individuals and Cisgender Individuals in the United Kingdom's Clinical Practice Research Datalink 1988–2019 (Without Imputation)

eTable 10. Overall and Cause-Specific Mortality Rate Ratios for Transgender and Gender Diverse Individuals Compared to Cisgender Individuals in the United Kingdom's Clinical Practice Research Datalink (Without Imputation)

eTable 11. Overall and Cause-Specific Mortality Rate Ratios for Transgender and Gender Diverse Individuals Compared to Cisgender Individuals in the United Kingdom's Clinical Practice Research Datalink (Only Patients Who Could Have Died During or After 1998)

eTable 12. Mortality Rate Ratios for Deaths Due to Select Causes of Death Among Transgender and Gender Diverse Individuals Compared to Cisgender Individuals in the United Kingdom's Clinical Practice Research Datalink (Without Imputation)

© 2023 Jackson SS et al. *JAMA Network Open.*

eTable 13. Mortality Rate Ratios for Deaths Due to Select Causes Among Transgender and Gender Diverse Individuals Compared to Cisgender Individuals in the United Kingdom's Clinical Practice Research Datalink (Only Patients Who Could Have Died During or After 1998)

eFigure. Flow Chart of Final Analysis Cohort Combining CPRD GOLD and CPRD Aurum

This supplemental material has been provided by the authors to give readers additional information about their work.

eAppendix. Supplemental Methods

A diagnosis of gender incongruence (formerly gender dysphoria, which refers to an individual's profound discomfort with the incongruence between experienced identity and assigned birth sex) is required for individuals to begin gender affirming treatment in most countries, including England. Some transgender and gender diverse (TGD) individuals take hormones or undergo gender affirming surgery (e.g., gender affirming care) to alleviate symptoms of gender incongruence. Some TGD individuals feel that neither the masculine nor feminine gender expression matches their identity (e.g. gender nonbinary persons) and may elect for varying levels of medical and surgical transition.[1] To receive gender affirming care within the National Health Services (NHS) system, adults must be referred by their general practitioner to one of seven gender identity clinics in England. Those <18 years of age must be referred to the Gender Identity Development Service and may be referred on to a gender identity clinic when approaching 18. Diagnoses and treatments received from the gender identity clinics are also transmitted back to the patient's primary care record.

We used diagnostic codes for gender incongruence to identify TGD individuals in CPRD (eTables 1–2). We excluded those with a diagnosis code for differences of sex characteristics (formerly disorders of sex development) (eTables 3–4). These TGD persons were then individually matched to 20 cis men and 20 cis women comprised of patients who have never had a record of gender incongruence or differences of sex characteristics with the following requirements: same year of birth (±1 year), same practice, cisgender control is registered at the time of TGD index date, and TGD person and cisgender control are registered no more than 1 year apart.

Sex assigned at birth for TGD patients was determined by the documentation of gender affirming therapies and surgeries, and sex-specific procedures listed in the primary care database, the linked Hospital Episode Statistics (HES) Admitted Patient Care and Outpatient Care files. The Admitted Patient Care covers the period April 1997 to June 2019 and contains records of all inpatient hospital admissions for patients at NHS hospitals in England. The Outpatient Care covers the period April 2003–June 2019 and contains records from outpatient appointments occurring in England. Medications used for gender affirming hormone therapy are listed in eTable 5 and gender affirming procedures are listed in eTable 6 for Read Terms in CPRD GOLD and eTable 7 for SNOMED terms in CPRD Aurum. Primary diagnoses were coded using the International Statistical Classification of Diseases, Tenth Revision (ICD-10), and procedures were coded using the Classification of Interventions and Procedures from the

© 2023 Jackson SS et al. *JAMA Network Open.*

Office of Population Census and Surveys codes version 4.4 (eTable 8). Patients with conflicting records were reviewed by a specialist (A.M.B.) who determined assigned sex at birth or recommended exclusion.

These methods enabled us to capture sex assigned at birth from 67% of TGD individuals. For the remaining unclassifiable TGD individuals we used multiple imputation with chained equations to impute missing sex assigned at birth. The results of the non-imputed analysis where sex assigned at birth was not imputed for TGD individuals with missing sex in addition to those with missing values for BMI, smoking status, or alcohol use were excluded are presented in Tables S9–S10. We identified 1330 transfeminine individuals, 892 transmasculine individuals, and 1095 TGD individuals whose sex assigned at birth was unknown matched to 68165 cisgender men and 68004 cisgender women from the CPRD GOLD and Aurum databases between 1988 to 2019.

We used multiple imputation with chained equations, as implemented in in PROC MI (SAS 9.4; SAS Institute Inc., Cary, NC) to impute missing values for sex assigned at birth and other variables with missing values (between 5–24% of BMI, smoking status, and alcohol use data were missing). We used age at index date, index year, height, weight, death, race/ethnicity, index of multiple deprivation, BMI, smoking status, and alcohol use to predict the missing values. We created 5 imputed datasets, analyzed them separately, and combined the results using Rubin’s formula for the variance as implemented in PROC MIANALYZE. We used PROC SURVEYFREQ and PROC MIANALYZE to obtain pooled frequencies and proportions.

© 2023 Jackson SS et al. *JAMA Network Open.*

**eTable 1.** Read Codes and Terms to Identify Transgender and Gender Diverse Individuals in CPRD GOLD	
**Read code**	**Read term**
1K4-00	Gender reassignment patient
Eu64.00	[X]Gender identity disorders
E22y400	Gender role disorder of adolescent or adult
Eu64y00	[X]Other gender identity disorders
Eu64200	[X]Gender identity disorder of childhood
Eu64z00	[X]Gender identity disorder, unspecified
Eu64000	[X]Transsexualism
1K-.00	Gender[a]
[a]Individuals with only this Read term in the medical record were required to additionally have evidence of gender affirming care (gender affirming hormone therapy or gender affirming surgery) to be included in the TGD group.	

**eTable 2**. SNOMED Codes and Terms to Identify Transgender and Gender Diverse Individuals in CPRD Aurum	
**SNOMED description ID**	**Term**
41693015	Operation for sexual transformation NOS
41693015	Other specified operation for sexual transformation
145857010	[X]Other gender identity disorders
370377016	Desire to become member of the opposite sex
2158979015	[X]Transsexualism
2158979015	Transsexual
2158979015	Trans-sexualism
2158979015	Trans-sexualism NOS
2158979015	Trans-sexuality with unspecified sexual history
2158981018	Male-to-female transsexual
2158982013	Female-to-male transsexual
2158983015	Surgically transgendered transsexual, male-to-female
2158984014	Surgically transgendered transsexual, female-to-male
2952273010	Trans-sexuality with heterosexual history
2952280012	Trans-sexuality with homosexual history
2952296010	Trans-sexuality with asexual history
2955531013	[X]Dual-role transvestism
2955541011	[X]Fetishistic transvestism
3298519013	Male to female transsexual person on hormone therapy
3298557013	Female to male transsexual person on hormone therapy
3317388016	Operations for sexual transformation
2003021000006114	Seen in gender identity clinic
2227021000000113	Referral to LGBT (lesbian, gay, bisexual and transgender) service[a]
2679981000000118	Non-binary gender
[a]Individuals with only this term in the medical record were required to additionally have evidence of gender affirming care (gender affirming hormone therapy or gender affirming surgery) to be included in the transgender cohort.	

© 2023 Jackson SS et al. *JAMA Network Open.*

**eTable 3**. Read Codes and Terms to Exclude Individuals With Variations of Sex Characteristics in CPRD GOLD	
**Read code**	**Read term**
PC7z.00	Indeterminate sex or pseudohermaphroditism NOS
PC7z000	Indeterminate sex NOS
PC7-00	Indeterminate sex and pseudohermaphroditism
C152814	Pseudohermaphrodite, male with adrenocortical disorder
PC71.00	Male pseudohermaphroditism
PC70	True hermaphroditism
PC72.00	Female pseudohermaphroditism
PC7z111	False hermaphrodite
PJyy100	46XX true hermaphrodite
K01w112	Wilms' tumour + nephrotic syndrome + pseudohermaphroditism

**eTable 4**. SNOMED Codes and Terms to Exclude Individuals With Variations of Sex Characteristics in CPRD Aurum	
**SNOMED description ID**	**Term**
15516019	Female pseudohermaphroditism
178565013	Male pseudohermaphroditism
315484010	Chimera 46XX/46XY, true hermaphrodite
315485011	46XX true hermaphrodite
354341016	Nephrotic syndrome with pseudohermaphroditism
354342011	Wilms' tumour + nephrotic syndrome + pseudohermaphroditism
356304015	Pseudohermaphrodite, male with adrenocortical disorder
356309013	Pseudohermaphrodite,female with adrenocortical disorder
377946011	False hermaphrodite
400865010	Indeterminate sex and pseudohermaphroditism
400865010	Indeterminate sex NOS
400865010	Indeterminate sex or pseudohermaphroditism NOS
400865010	Pseudohermaphrodite NOS
478925016	True hermaphroditism
3047788013	Intersex NEC
2301891000000112	Operations for disorders of sex development
2301891000000112	Other specified operations for disorders of sex development
2301891000000112	Unspecified operations for disorders of sex development

© 2023 Jackson SS et al. *JAMA Network Open.*

eTable 5. Medications Extracted From the Medical Record Used to Identify Sex Assigned at Birth Among Transgender and Gender Diverse Participants in CPRD Aurum and GOLD

Product name	Indication	Assigned sex at birth
Andropatch	Androgen	Female
Intrinsa		
Mesterolone		
Nebido		
Primoteston		
Proscar		
Restandol		
Sustanon		
Testim		
Testogel		
Testosterone		
Tostran		
Virormone		
5-alpha reductase inhibitor	Anti-Androgen	Male
Androcur		
Bicalutamide		
Cyprostat		
Cyproterone		
Dutasteride		
Finasteride		
Flutamide		
Spironolactone		
Letrozole	Aromatase inhibitor	Female
Tamoxifen		
Climaval	Estrogen	Male
Conjugated oestrogens		
Elleste		
Estraderm		
Estradiol		
Estradot		
Ethinylestradiol		
Evorel		
Femoston		
Femseven		
Hormonin		
Oestrogel		
Ortho dienoestrol		
Premarin		
Prempak		
Progynova		
Sandrena		
Trisequins		
Zumenon		
Crinone	Progestin	Female
Depo-provera		
Etonogestrel		
Implanon		
Nexplanon		
Norethisterone		
Provera		
Utovlan		

© 2023 Jackson SS et al. *JAMA Network Open.*

Medroxyprogesterone<br>Provera	Progestogens	Male

**eTable 6**. Read Codes and Terms to Identify Sex Assigned at Birth for Transgender and Gender Diverse Individuals in CPRD GOLD		
**Read code**	**Read Term**	**Assigned sex at birth**
ZV6G100	[V]Acquired absence of breast(s)	Female
ZV50111	[V]Breast augmentation	Female
7E04511	Abdominal hysterectomy & bilateral salpingoophorectomy (BSO)	Female
7E04500	Abdominal hysterectomy and bilateral salpingoophorectomy	Female
7E04800	Abdominal hysterectomy and left salpingoophorectomy	Female
7130800	Bilateral mastectomy	Female
7E10200	Bilateral oophorectomy NEC	Female
7E10000	Bilateral salpingoophorectomy	Female
G860.11	Elephantiasis-postmastectomy	Female
159B.00	H/O: bilateral oophorectomy	Female
7130511	Halsted radical mastectomy	Female
7E05600	Lap assist vag hysterectomy with bilat salpingo-oophorectomy	Female
7E04B00	Lapar total abdominal hysterect bilat salpingo-oophorectomy	Female
6753	Mastectomy counselling	Female
7130900	Modified radical mastectomy	Female
7E12200	Oophorectomy NEC	Female
7131.11	Other mastectomy operations	Female
7130y00	Other specified total mastectomy	Female
7131100	Partial mastectomy NEC	Female
G860	Postmastectomy lymphoedema	Female
7130512	Radical mastectomy	Female
7130500	Radical mastectomy including axillary lymph nodes	Female
7130600	Simple mastectomy	Female
7130A00	Skin-sparing mastectomy	Female
7130400	Subcutaneous mastectomy	Female
7130700	Subcutaneous mastectomy for gynaecomastia	Female
7E04512	TAH - total abdom hysterectomy & bilateral salpingoophorect	Female
7130000	Total mastectomy & excision pectoral muscs & part chest wall	Female
7130100	Total mastectomy and excision of both pectoral muscles NEC	Female
7130200	Total mastectomy and excision of pectoralis minor muscle	Female
7130300	Total mastectomy NEC	Female
7130z00	Total mastectomy NOS	Female
7130	Total mastectomy operations	Female

**eTable 7**. SNOMED Codes and Terms to Identify Sex Assigned at Birth for Transgender and Gender Diverse Individuals in CPRD Aurum		
**SNOMED description ID**	**Term**	**Assigned sex at birth**
371333019	[V]Acquired absence of breast(s)	Female
184451014	Abdominal hysterectomy	Female
184453012	Abdominal hysterectomy & bilateral salpingoophorectomy (BSO)	Female
2900473014	Abdominal hysterectomy & excision of periuterine tissue NEC	Female
184453012	Abdominal hysterectomy and bilateral salpingoophorectomy	Female
443763014	Abdominal hysterectomy and left salpingoopherectomy	Female

© 2023 Jackson SS et al. *JAMA Network Open.*

443763014	Abdominal hysterectomy and left salpingoophorectomy	Female
443762016	Abdominal hysterectomy and right salpingoopherectomy	Female
2474692011	Abdominal hysterectomy with conservation of ovaries	Female
870341000006119	Bilat. salpingo-oophorectomy	Female
273170011	Bilateral microvascular orchidopexy	Female
127631019	Bilateral oophorectomy NEC	Female
49911018	Bilateral salpingoophorectomy	Female
451138019	Bonney abdominal hysterectomy	Female
474933010	Extended radical mastectomy	Female
858661000006111	Extended simple mastectomy	Female
1485111017	H/O: bilateral oophorectomy	Female
252126010	H/O: hysterectomy	Female
474903015	Halsted radical mastectomy	Female
393622010	Heaney vaginal hysterectomy	Female
2817411010	Lap assist vag hysterectomy with bilat salpingo-oophorectomy	Female
2534190016	Lapar total abdominal hysterect bilat salpingo-oophorectomy	Female
1485111017	H/O: bilateral oophorectomy	Female
252126010	H/O: hysterectomy	Female
474903015	Halsted radical mastectomy	Female
393622010	Heaney vaginal hysterectomy	Female
2817411010	Lap assist vag hysterectomy with bilat salpingo-oophorectomy	Female
2534190016	Lapar total abdominal hysterect bilat salpingo-oophorectomy	Female
355049018	Laparoscopic hysterectomy	Female
2773128015	Laparoscopic subtotal hysterectomy	Female
1233920014	Laparoscopic vaginal hysterectomy	Female
1485111017	H/O: bilateral oophorectomy	Female
252126010	H/O: hysterectomy	Female
474903015	Halsted radical mastectomy	Female
393622010	Heaney vaginal hysterectomy	Female
1573171000006118	Laparoscopic-assist vaginal hysterec bilat salpingo-oophorectomy	Female
444030018	Left oophorectomy NEC	Female
273903010	Left salpingoophorectomy	Female
2158157014	Modified radical mastectomy	Female
2767711012	Other mastectomy operations	Female
266409013	Other specified total mastectomy	Female
106993015	Partial mastectomy NEC	Female
451138019	Radical abdominal hysterectomy	Female
184456016	Radical hysterectomy	Female
1894251000006114	Radical hysterectomy with bilateral salpingo-oophorectomy	Female
2358601000000119	Radical hysterectomy with conservation of ovaries	Female
1479045012	Radical mastectomy	Female
410874017	Radical mastectomy including axillary lymph nodes	Female
197188014	Radical vaginal hysterectomy	Female
444029011	Right oophorectomy NEC	Female
273900013	Right salpingoophorectomy	Female
871011000006111	Salango - oophorotomy	Female
184484014	Salpingoophorectomy NEC	Female
184484014	Salpingoophorectomy remaining solitary fallop tube and ovary	Female
486325012	Schauta radical vaginal hysterectomy	Female
266409013	Simple mastectomy	Female
2694270019	Skin-sparing mastectomy	Female
116564019	Subcutaneous mastectomy	Female
870101000006119	Subtotal abdom. hysterectomy	Female

© 2023 Jackson SS et al. *JAMA Network Open.*

273717011	Subtotal abdominal hysterectomy	Female
2358481000000110	Subtotal abdominal hysterectomy & left salpingo-oophorectomy	Female
1894281000006118	Subtotal abdominal hysterectomy and left salpingo-oophorectomy	Female
1894271000006116	Subtotal abdominal hysterectomy and right salpingo-oophorectomy	Female
1894241000006112	Subtotal abdominal hysterectomy with bilat salpingo-oophorectomy	Female
273717011	Subtotal abdominal hysterectomy with conservation of ovaries	Female
2358381000000117	Subtotl abdominal hysterectomy & bilat salpingo-oophorectomy	Female
2358441000000119	Subtotl abdominal hysterectomy & right salpingo-oophorectomy	Female
184453012	TAH - Tot abdom hysterectomy and BSO - bilat salpingophorect	Female
1220269014	TAH - total abdom hysterectomy & bilateral salpingoophorect	Female
184452019	Total abdominal hysterectomy NEC	Female
628771000000110	Total abdominal hysterectomy with conservation of ovaries	Female
853791000006118	Total hysterectomy	Female
851411000006118	Total hysterectomy & BSO	Female
474933010	Total mastectomy & excision pectoral muscs & part chest wall	Female
355751013	Total mastectomy and excision of both pectoral muscles NEC	Female
1489357016	Total mastectomy and excision of pectoralis minor muscle	Female
1221494019	Total mastectomy NEC	Female
1221494019	Total mastectomy NOS	Female
1221494019	Total mastectomy operations	Female
870391000006111	Unilat.salpingo-oophorectomy	Female
55338015	Unilateral oophorectomy NEC	Female
184484014	Unilateral salpingoophorectomy NEC	Female
393622010	Vaginal hysterectomy	Female
2899369019	Vaginal hysterectomy and excision of periuterine tissue NEC	Female
1894971000006114	Vaginal hysterectomy and left salpingo-oophorectomy	Female
2358521000000110	Vaginal hysterectomy and right salpingo-oophorectomy	Female
393622010	Vaginal hysterectomy NEC	Female
2474693018	Vaginal hysterectomy with conservation of ovaries	Female
393622010	Ward vaginal hysterectomy	Female
451139010	Wertheim hysterectomy	Female
134108016	Amputation of penis	Male
134108016	Amputation of penis NOS	Male
273169010	Bevan bilateral orchidopexy	Male
273144010	Bilateral inguinal orchidectomy	Male
46639019	Bilateral mastectomy	Male
1480776010	Bilateral orchidectomy	Male
1480776010	Bilateral orchidectomy NOS	Male
273169010	Bilateral orchidopexy	Male
273169010	Bilateral orchidopexy NOS	Male
273147015	Bilateral scrotal orchidectomy	Male
273141019	Bilateral subcapsular orchidectomy	Male
273145011	Bilateral total inguinal orchidectomy	Male
273143016	Bilateral total orchidectomy, unspecified method	Male
273146012	Bilateral total scrotal orchidectomy	Male
273175018	First stage bilateral orchidopexy	Male
443236014	First stage unilateral orchidopexy	Male

© 2023 Jackson SS et al. *JAMA Network Open.*

251866010	H/O: orchidectomy	Male
868781000006115	Incision of testis-orchidotomy	Male
273169010	Ombredanne bilateral orchidopexy	Male
1481753016	Ombredanne unilateral orchidopexy	Male
1481753016	One stage unilateral orchidopexy	Male
1481753016	Other one stage bilateral orchidopexy	Male
134108016	Other specified amputation of penis	Male
1480776010	Other specified bilateral orchidectomy	Male
3512849014	Other specified unilateral orchidopexy	Male
273176017	Second stage bilateral orchidopexy	Male
443241018	Second stage unilateral orchidopexy	Male
868641000006115	Testis excision/orchidectomy	Male
273304010	Total amputation of penis	Male
2883990010	Unilateral inguinal orchidectomy	Male
141585012	Unilateral microvascular orchidopexy	Male
868661000006116	Unilateral orchidectomy	Male
354273013	Unilateral scrotal orchidectomy	Male
354273013	Unilateral subcapsular orchidectomy	Male
354273013	Unilateral total orchidectomy - unspecified	Male

**eTable 8**. Sex-Specific Procedures Extracted From the Hospital Episode Statistics Admitted Patient Care and Outpatient Care Files Used to Identify Sex Assigned at Birth Among Transgender and Gender Diverse Individuals			
**OPCS Code**	**Procedure**	**General Classification**	**Sex Assigned At Birth**
B27.x	Total excision of breast, total mastectomy and excision of both pectoral muscles and part of chest wall, total mastectomy and excision of both pectoral muscles NEC, total mastectomy and excision of pectoralis minor muscle, total mastectomy NEC, subcutaneous mastectomy, skin sparing mastectomy, mastectomy, other specified total excision of breast, unspecified total excision of breast	Mastectomy	Female
B31.1	Reduction mammoplasty	Other plastic operations on breast	Female
Q01.1, Q01.2, Q01.3, Q01.4, Q01.8, Q01.9	Amputation of cervix uteri, wedge excision of cervix uteri and suture HFQ, excision of lesion of cervix uteri, large loop excision of transformation zone, other specified excision of cervix uteri, unspecified excision of cervix uteri	Excision of cervix uteri	Female
Q02.1, Q02.2, Q02.3, Q02.4, Q02.8, Q02.9	Avulsion of lesion of cervix uteri, laser destruction of lesion of cervix uteri, cauterisation of lesion of cervix uteri, cryotherapy to lesion of cervix uteri, other specified destruction of lesion of cervix uteri, unspecified destruction of lesion of cervix uteri	Destruction of lesion of cervix uteri	Female
Q03.1, Q03.2, Q03.3, Q03.4, Q03.5, Q03.8, Q03.9	Knife cone biopsy of cervix uteri, laser cone biopsy of cervix uteri, cone biopsy of cervix uteri NEC, punch biopsy of cervix uteri, ring biopsy of cervix uteri, other specified biopsy of cervix uteri, unspecified biopsy of cervix uteri	Biopsy of cervix uteri	Female
Q05.1, Q05.2., Q05.8, Q05.9	Repair of cervix uteri NEC, dilation of cervix uteri, other specified other operations on cervix uteri, unspecified other operations on cervix uteri	Other operations on cervix uteri	Female
Q07.1, Q07.2, Q07.3, Q07.4, Q07.5, Q07.6, Q07.8, Q07.9	Abdominal hysterocolpectomy and excision of periuterine, abdominal hysterectomy and excision of periuterine tissue NEC, abdominal hysterocolpectomy NEC, total abdominal hysterectomy NEC, subtotal abdominal hysterectomy, excision of accessory uterus, other	Hysterectomy	Female

© 2023 Jackson SS et al. *JAMA Network Open.*

	specified abdominal excision of uterus, unspecified abdominal excision of uterus		
Q08.1, Q08.2, Q08.3, V08.8, Q08.9	Vaginal hysterectomy and excision of periuterine tissue NEC, vaginal hysterocolpectomy NEC, other specified vaginal excision of uterus, unspecified vaginal excision of uterus		
Q09.1, Q09.2, Q09.3, Q09.3, Q09.4, Q09.5, Q09.6, Q09.8, Q09.9	Open removal of products of conception from uterus, open myomectomy, open excision of lesion of uterus NEC, open biopsy of lesion of uterus, metroplasty, Incision of uterus NEC, other specified other open operations on uterus, unspecified other open operations on uterus	Other open operations on uterus	Female
Q10.1, Q10.2, Q10.3, Q10.8, Q10.9	Dilation of cervix uteri and curettage of products of conception from uterus, curettage of products of conception from uterus NEC,<br>dilation of cervix uteri and curettage of uterus NEC, other specified curettage of uterus, unspecified curettage of uterus	Curettage of uterus	Female
Q11.1, Q11.2, Q11.3, Q11.4, Q11.5, Q11.6, Q1.8, Q11.9	Vacuum aspiration of products of conception from uterus NEC, dilation of cervix uteri and evacuation of products of conception from uterus NEC,<br>evacuation of products of conception from uterus NEC,<br>extraction of menses, vacuum aspiration of products of conception from uterus using rigid cannula, vacuum aspiration of products of conception from uterus using flexible cannula, other specified other evacuation of contents of uterus, unspecified other evacuation of contents of uterus	Other evacuation of contents of uterus	Female
Q12.1, Q12.2, Q12.3, Q12.4, Q12.8, Q12.9	Introduction of intrauterine contraceptive device, replacement of intrauterine contraceptive device, removal of displaced intrauterine contraceptive device NEC, removal of intrauterine contraceptive device NEC, other specified intrauterine contraceptive device, unspecified intrauterine contraceptive device	Intrauterine contraceptive device	Female

© 2023 Jackson SS et al. *JAMA Network Open.*

Q13.x	Transfer of embryo to uterus NEC, intracervical artificial insemination, intrauterine artificial insemination, intrauterine insemination with superovulation using partner sperm, intrauterine insemination with superovulation using donor sperm, intrauterine insemination without superovulation using partner sperm, intrauterine insemination without superovulation using donor sperm, other specified introduction of gametes into uterine cavity, unspecified introduction of gametes into uterine cavity	Introduction of gamete into uterine cavity	Female
Q14.1, Q14.2, Q14.3, Q14.4, Q14.5, Q14.6, Q14.8, Q14.9	Intra-amniotic injection of prostaglandin, intra-amniotic injection of abortifacient NEC, extra-amniotic injection of prostaglandin, extra-amniotic injection of abortifacient NEC, insertion of prostaglandin pessary, insertion of abortifacient pessary NEC, other specified introduction of abortifacient into uterine cavity, unspecified introduction of abortifacient into uterine cavity	Introduction of abortifacient into uterine cavity	Female
Q15.1, Q15.2, Q15.3, Q15.4, Q15.8, Q15.9	Introduction of radioactive substance into uterine cavity, introduction of therapeutic substance into uterine cavity NEC, injection into uterine cavity NEC, removal of therapeutic substance from uterine cavity, other specified introduction of other substance into uterine cavity, unspecified introduction of other substance into uterine cavity	Introduction of other substance into uterine cavity	Female
Q16.1, Q16.2, Q16.3, Q16.4, Q16.5, Q16.6, Q16.8, Q16.9	Vaginal excision of lesion of uterus, balloon ablation of endometrium, Microwave ablation of endometrium NEC, free circulating saline ablation of endometrium, radiofrequency ablation of endometrium, photodynamic ablation of endometrium, other specified other vaginal operations on uterus, unspecified other vaginal operations on uterus	Other vaginal operations on uterus	Female
Q17.x	Endoscopic resection of lesion of uterus, endoscopic cauterisation of lesion of uterus, endoscopic cryotherapy to lesion of uterus, endoscopic destruction of lesion of uterus NEC, endoscopic metroplasty, endoscopic microwave ablation of endometrium, Endoscopic balloon ablation of endometrium, other specified therapeutic endoscopic	Therapeutic endoscopic operations on uterus	Female

© 2023 Jackson SS et al. *JAMA Network Open.*

	operations on uterus, unspecified therapeutic endoscopic operations on uterus		
Q18.1, Q18.8, Q18.9	Diagnostic endoscopic examination of uterus and biopsy of lesion of uterus, other specified diagnostic endoscopic examination of uterus, unspecified diagnostic endoscopic examination of uterus	Diagnostic endoscopic examination of uterus	Female
Q19.1, Q19.2, Q19.3	Connection of uterus to vagina, other specified plastic operations on uterus, unspecified plastic operations on uterus	Plastic operations on uterus	Female
Q20.1, Q20.2, Q20.3, Q20.4, Q20.5, Q20.6 Q20.8, Q20.9	Freeing of adhesions of uterus, biopsy of lesion of uterus NEC, manual manipulation of uterus, vaginofixation of uterus, exploration of uterus NEC, focused ultrasound to lesion of uterus, other specified other operations on uterus, unspecified other operations on uterus	Other operations on uterus	Female
Q21.1, Q21.8 , Q21.9	Transmyometrial transfer of embryo to uterus, other specified other introduction of gametes into uterine cavity, unspecified other introduction of gametes into uterine cavity	Other introduction of gamete into uterine cavity	Female
Q22.1, Q22.2, Q22.3, Q22.8, Q22.9	Bilateral salpingoophorectomy, Bilateral salpingectomy NEC, Bilateral oophorectomy NEC, other specified bilateral excision of adnexa of uterus, unspecified bilateral excision of adnexa of uterus	Bilateral excision of adnexa of uterus	Female
Q23.1, Q23.2, Q23.3, Q23.4, Q23.5, Q23.6, Q23.8, Q23.9	Unilateral salpingoophorectomy NEC, salpingoophorectomy of remaining solitary fallopian tube and ovary, unilateral salpingectomy NEC, salpingectomy of remaining solitary fallopian tube NEC, unilateral oophorectomy NEC, oophorectomy of remaining solitary ovary NEC, other specified unilateral excision of adnexa of uterus, unspecified unilateral excision of adnexa of uterus	Unilateral excision of adnexa of uterus	Female
Q24.1, Q24.3	Salpingoophorectomy NEC, oophorectomy NEC	Other excision of adnexa of uterus	Female
Q25.1, Q25.8, Q25.9	Excision of lesion of fallopian tube, other specified partial excision of fallopian tube, unspecified partial excision of fallopian tube	Partial excision of fallopian tube	Female
Q26.1, Q26.2, Q26.3, Q26.8, Q26.9	Insertion of tubal prosthesis into fallopian tube, revision of tubal prosthesis in fallopian tube, removal of tubal prosthesis from fallopian tube, other specified placement of prosthesis in fallopian tube, unspecified placement of prosthesis in fallopian tube	Placement of prosthesis in fallopian tube	Female

© 2023 Jackson SS et al. *JAMA Network Open.*

Q27.1, Q27.2, Q27.8, Q27.9	Open bilateral ligation of fallopian tubes, open bilateral clipping of fallopian tubes, other specified open bilateral occlusion of fallopian tubes, unspecified open bilateral occlusion of fallopian tubes	Open bilateral occlusion of fallopian tubes	Female
Q28.1, Q28.2, Q28.3, Q28.4, Q28.8, Q28.9	Open ligation of remaining solitary fallopian tube, open ligation of fallopian tube NEC, open clipping of remaining solitary fallopian tube, open clipping of fallopian tube NEC, other specified other open occlusion of fallopian tube, unspecified other open occlusion of fallopian tube	Other open occlusion of fallopian tube	Female
Q29.1, Q29.2, Q29.8, Q29.9	Reanastomosis of fallopian tube NEC, open removal of clip from fallopian tube NEC, other specified open reversal of female sterilization, unspecified open reversal of female sterilisation	Open reversal of female sterilisation	Female
Q30.1, Q30.2, Q30.3, Q30.4, Q30.5, Q30.8, Q30.9	Reconstruction of fallopian tube, replantation of fallopian tube, anastomosis of fallopian tube NEC, salpingostomy, suture of fallopian tube, other specified other repair of fallopian tube, unspecified other repair of fallopian tube	Other incision of fallopian tube and repair fallopian tube	Female
Q31.1, Q31.2, Q31.8, Q31.9	Removal of products of conception from fallopian tube, drainage of fallopian tube, other specified incision of fallopian tube, unspecified incision of fallopian tube	Incision of fallopian tube	Female
Q32.1, Q32.2, Q32.3, Q32.8, Q32.9	Excision of fimbria, burying of fimbria in wall of uterus, excision of hydatid of Morgagni, other specified operations on fimbria, unspecified operations on fimbria	Operations on fimbria	Female
Q34.1, Q34.2, Q34.3, Q34.4, Q34.8, Q34.9	Open freeing of adhesions of fallopian tube, open biopsy of fallopian tube, open dilation of fallopian tube, exploration of fallopian tube, other specified other open operations on fallopian tube, unspecified other open operations on fallopian tube	Other open operations on fallopian tube	Female
Q35.1, Q35.2, Q35.3, Q35.4, Q35.8, Q35.9	Endoscopic bilateral cauterisation of fallopian tubes, endoscopic bilateral clipping of fallopian tubes, endoscopic bilateral ringing of fallopian tubes, endoscopic bilateral placement of intrafallopian implants, other specified endoscopic bilateral occlusion of fallopian tubes, unspecified endoscopic bilateral occlusion of fallopian tubes	Endoscopic bilateral occlusion of fallopian tubes	Female

© 2023 Jackson SS et al. *JAMA Network Open.*

Q36.1, Q36.2, Q36.8, Q36.9	Endoscopic occlusion of remaining solitary fallopian tube, endoscopic placement of intrafallopian implant into remaining solitary fallopian tube, other specified other endoscopic occlusion of fallopian tube, unspecified other endoscopic occlusion of fallopian tube	Other endoscopic occlusion of fallopian tube	Female
Q37.1, Q37.8, Q37.9	Endoscopic removal of clip from fallopian tube, other specified endoscopic reversal of female sterilization, unspecified endoscopic reversal of female sterilisation	Endoscopic reversal of female sterilisation	Female
Q38.1, Q38.2, Q38.3, Q38.8, Q38.9	Endoscopic freeing of adhesions of fallopian tube, endoscopic injection into fallopian tube, endoscopic intrafallopian transfer of gametes, other specified other therapeutic endoscopic operations on fallopian tube, unspecified other therapeutic endoscopic operations on fallopian tube	Other therapeutic endoscopic operations on fallopian tube	Female
Q39.1, Q39.8, Q39.9	Diagnostic endoscopic examination of fallopian tube and biopsy of lesion of fallopian tube, Other specified diagnostic endoscopic examination of fallopian tube, Unspecified diagnostic endoscopic examination of fallopian tube	Diagnostic endoscopic examination of fallopian tube	Female
Q41.x	Salpingography, hydrotubation of fallopian tube, Dye test of fallopian tube, insufflation of fallopian tube, operations to ensure patency of fallopian tube NEC, recanalisation of fallopian tube, aspiration of fallopian tube, other specified other operations on fallopian tube, unspecified other operations on fallopian tube	Other operations on fallopian tube	Female
Q43.1, Q43.2, Q43.3, Q43.8, Q43.9	Excision of wedge of ovary, excision of lesion of ovary, marsupialisation of lesion of ovary, other specified partial excision of ovary, unspecified partial excision of ovary	Partial excision of ovary	Female
Q44.1, Q44.8, Q44.9	Open cauterisation of lesion of ovary, other specified open destruction of lesion of ovary, unspecified open destruction of lesion of ovary	Open destruction of lesion of ovary	Female
Q45.1, Q45.2, Q45.3, Q45.4, Q45.8, Q45.9	Replantation of ovary, fixation of ovary NEC, suture of ovary, suture of rupture of corpus luteum, other specified repair of ovary, unspecified repair of ovary	Repair of ovary	Female
Q47.1, Q47.2, Q47.3, Q47.4, Q47.8, Q47.9	Transposition of ovary, open freeing of adhesions of ovary, open biopsy of lesion of ovary, open drainage of cyst	Other open operations on ovary	Female

© 2023 Jackson SS et al. *JAMA Network Open.*

	of ovary, other specified other open operations on ovary, unspecified other open operations on ovary		
Q48.1, Q48.2, Q48.3, Q48.4, Q48.8, Q48.9	Endoscopic transurethral ultrasound directed oocyte recovery, endoscopic transvesical oocyte recovery, laparoscopic oocyte recovery, transvaginal oocyte recovery, other specified oocyte recovery, unspecified oocyte recovery	Oocyte recovery	Female
Q49.1, Q49.2, Q49.2, Q49.4, Q49.8, Q49.9	Endoscopic extirpation of lesion of ovary NEC, endoscopic freeing of adhesions of ovary, endoscopic drainage of cyst of ovary, endoscopic drilling of ovary, other specified therapeutic endoscopic operations on ovary, unspecified therapeutic endoscopic operations on ovary	Therapeutic endoscopic operations on ovary	Female
Q50.1, Q50.8, Q50.9	Diagnostic endoscopic examination of ovary and biopsy of lesion of ovary, other specified diagnostic endoscopic examination of ovary, unspecified diagnostic endoscopic examination of ovary	Diagnostic endoscopic examination of ovary	Female
Q51.1, Q51.8, Q51.9	Transvaginal ultrasound guided aspiration of ovarian cyst, other specified other operations on ovary, unspecified other operations on ovary	Other operations on ovary	Female
Q52.1, Q52.3, Q52.3, Q52.8, Q52.9	Excision of lesion of broad ligament of uterus, destruction of lesion of broad ligament of uterus, shortening of broad ligament of uterus, other specified operations on broad ligament of uterus, unspecified operations on broad ligament of uterus	Operations on broad ligament of uterus	Female
Q54.x	Suspension of uterus NEC,<br>Plication of round ligament of uterus, division of uteropelvic ligament,<br>Suspension of uterus using mesh NEC,<br>Sacrohysteropexy, infracoccygeal hysteropexy, total removal of prosthetic material from previous suspension of uterus, other specified operations on other ligament of uterus, unspecified operations on other ligament of uterus	Operations on other ligament of uterus	Female
Q55.3, Q55.4	Papanicolau smear NEC<br>colposcopy of cervix,	Other examination of female genital tract	
Q56.1, Q56.2, Q56.8, Q56.9	fertility investigation of female NEC,<br>Fertiloscopy, other specified other operations on female genital tract, unspecified other operations on female genital tract	Other operations on female genital tract	Female

© 2023 Jackson SS et al. *JAMA Network Open.*

Q57.8, Q57.9	Other specified other operations on other ligament of uterus, unspecified other operations on other ligament of uterus	Other operations on other ligament of uterus	Female
Q58.x	Delivery of Terminated Fetus	Delivery of terminated Fetus	Female
N01.1, N01.2, N01.3, N01.8, N01.9	Excision of scrotum, excision of lesion of scrotum, destruction of lesion of scrotum, other specified extirpation of scrotum, unspecified extirpation of scrotum	Extirpation of scrotum	Male
N03.1, N03.2, N03.3, N03.4, N03.5, N03.6, N03.8, N03.9	Biopsy of lesion of scrotum, drainage of scrotum, suture of scrotum, exploration of scrotum, removal of foreign body from scrotum, reconstruction of scrotum, other specified other operations on scrotum, unspecified other operations on scrotum	Other operations on scrotum	Male
N05.1, N05.2, N05.3, N05.8, N05.9	Bilateral subcapsular orchidectomy, bilateral orchidectomy NEC, bilateral inguinal orchidectomy, other specified bilateral excision of testes, unspecified bilateral excision of testes	Bilateral excision of testes	Male
N06.1, N06.2, N06.3, N06.4, N06.5, N06.6, N06.8, N06.9	Subcapsular orchidectomy NEC, excision of aberrant testis, orchidectomy NEC, excision of testicular appendage, division of cremaster, inguinal orchidectomy NEC, other specified other excision of testis, unspecified other excision of testis	Other excision of testis	Male
N07.1, N07.2, N07.8, N07.9	Excision of lesion of testis, destruction of lesion of testis, other specified extirpation of lesion of testis, unspecified extirpation of lesion of testis	Extirpation of lesion of testis	Male
N08.1, N08.2, N08.3, N08.4,	Bilateral microvascular transfer of testes to scrotum, one stage bilateral orchidopexy NEC, first stage bilateral orchidopexy, second stage bilateral orchidopexy	Bilateral placement of testes in scrotum	Male
N09.2, N09.3, N09.4,	One stage orchidopexy NEC, first stage orchidopexy NEC, second stage orchidopexy NEC	Unspecified other placement of testis in scrotum	Male
N15.x	Bilateral epididymectomy, unilateral epididymectomy, excision of lesion of epididymis, drainage of epididymis, biopsy of lesion of epididymis, aspiration of lesion of epididymis, epididymovasostomy, other specified operations on epididymis, unspecified operations on epididymis	Operations on epididymis	Male

© 2023 Jackson SS et al. *JAMA Network Open.*

N17.1, N17.2, N17.8, N17.9	Bilateral vasectomy, ligation of vas deferens NEC, other specified excision of vas deferens, unspecified excision of vas deferens	Excision of vas deferens	Male
N18.1, N18.2, N18.8, N18.9	Reversal of bilateral vasectomy, suture of vas deferens NEC, other specified repair of spermatic cord, unspecified repair of spermatic cord	Repair of spermatic cord	Male
N19.1, N19.2, N19.8, N19.9	Ligation of varicocele, embolisation of varicocele, other specified operations on varicocele, unspecified operations on varicocele	Operations on varicocele	Male
N20.1, N20.2, N20.3, N20.4, N20.5, N20.8, N20.9	Excision of lesion of spermatic cord, biopsy of spermatic cord drainage of spermatic cord, vasotomy, vasography, other specified other operations on spermatic cord, unspecified other operations on spermatic cord	Other operations on spermatic cord	Male
N22.1, N22.2, N22.3, N22.4, N22.5, N22.8, N22.9	Excision of seminal vesicle, Incision of seminal vesicle, seminal vesiculography, transrectal needle biopsy of seminal vesicle, resection of ejaculatory duct, other specified operations on seminal vesicle, unspecified operations on seminal vesicle	Operations on seminal vesicle	Male
N26.1, N26.2, N26.8, N26.9	Total amputation of penis, partial amputation of penis, other specified amputation of penis, unspecified amputation of penis	Amputation of penis	Male
N30.1, N30.2, N30.3, N30.4, N30.5, N30.6, N30.8, N30.9	Prepuceplasty, freeing of adhesions of prepuce, circumcision, dorsal slit of prepuce, stretching of prepuce, manual reduction of prepuce, other specified operations on prepuce, unspecified operations on prepuce	Operations on prepuce	Male
N34.1, N34.2, N34.3, N34.4, N34.5, N34.6	Fertility investigation of male NEC, collection of sperm NEC, male colposcopy, microsurgical epididymal sperm aspiration, percutaneous epididymal sperm aspiration, testicular sperm extraction	Other operations on male genital tract	Male
P03.1, P03.2, P03.3, P03.4, P03.5, P03.8, P03.9	Excision of Bartholin gland, marsupialization of Bartholin gland, excision of lesion of Bartholin gland, drainage of Bartholin gland, operations on Bartholin duct, other specified operations on Bartholin gland, unspecified operations on Bartholin gland	Operations on Bartholin gland	Female
Rxx.x	Pregnancy related Codes	Childbirth	Female
X15.1	Combined operations for transformation from male to female	Transwoman gender affirming procedure	Male
X15.2	Combined operations for transformation from female to male	Transman gender affirming procedure	Female

© 2023 Jackson SS et al. *JAMA Network Open.*

Z43.4, Z43.5, Z43.6	Spermatic cord, Seminal vesicle, Male perineum	Male genital organ	Male
Z44.2, Z44.6	Bartholin gland, Pouch of Douglas	Vagina	Female
Z45	Uterus	Uterus	Female
Z46.1, Z46.2, Z46.3, Z46.4	Fimbria, Fallopian tube, Ovary, Broad ligament of uterus	Other female genital tract	Female

© 2023 Jackson SS et al. *JAMA Network Open.*

**eTable 9.** Characteristics of Transgender and Gender Diverse Individuals and Cisgender Individuals in the United Kingdom's Clinical Practice Research Datalink 1988–2019 (Without Imputation)					
	**Transfeminine individuals n=1330**	**Transmasculine individuals n=892**	**TGD, unknown assigned sex at birth n=1095**	**Cisgender men n=68165**	**Cisgender women n=68004**
**Age,** mean (SD)	37.8 (13.8)	27.4 (10.0)	33.9 (13.1)	33.6 (13.2)	33.5 (13.2)
**Index year**, median (IQR)	2009 (14)	2015 (9)	2010 (11)	2011 (13)	2011 (13)
**Body mass index**, n (%)					
Underweight	68 (5.1)	26 (2.9)	48 (4.4)	1708 (2.5)	2544 (3.7)
Normal	530 (39.8)	309 (34.6)	385 (35.2)	20979 (30.8)	26812 (39.4)
Overweight	336 (25.3)	199 (22.3)	234 (21.4)	18117 (26.6)	15128 (22.2)
Obese	261 (19.6)	231 (25.9)	220 (20.1)	10789 (15.8)	13854 (20.4)
Missing	135 (10.2)	127 (14.2)	208 (19.0)	16572 (24.3)	9666 (14.2)
**Smoking Status,** n (%)					
Never	426 (32.0)	311 (34.9)	347 (31.7)	24205 (35.5)	27758 (40.8)
Former	458 (34.4)	288 (32.3)	269 (24.6)	16685 (24.5)	19172 (28.2)
Current	369 (27.7)	249 (27.9)	411 (37.5)	21162 (31.0)	17554 (25.8)
Missing	77 (5.8)	44 (4.9)	68 (6.2)	6113 (9.0)	3520 (5.2)
**Alcohol use**, n (%)					
Never	76 (5.7)	79 (8.9)	91 (8.3)	3631 (5.3)	5321 (7.8)
Former	55 (4.1)	49 (5.5)	78 (7.1)	2157 (3.2)	3023 (4.4)
Current	1015 (76.3)	594 (66.6)	710 (64.8)	47056 (69.0)	47475 (69.8)
Missing	184 (13.8)	170 (19.1)	216 (19.7)	15321 (22.5)	12185 (17.9)
**Race/ethnicity**,[a] n (%)					
Asian	16 (1.2)	10 (1.1)	13 (1.2)	2384 (3.5)	3055 (4.5)
Black	12 (0.9)	19 (2.1)	5 (0.5)	1754 (2.6)	2343 (3.4)
White	1010 (75.9)	683 (76.6)	770 (70.3)	41174 (60.4)	45534 (67.0)
Other	45 (3.4)	39 (4.4)	33 (3.0)	1944 (2.9)	2435 (3.6)
Unknown or Another race	247 (18.6)	141 (15.8)	274 (25.0)	20909 (30.7)	14637 (21.5)
**Index of Multiple Deprivation**,[b] n (%)					
1st Quintile	172 (12.9)	105 (11.8)	132 (12.1)	8346 (12.2)	8310 (12.2)
2nd Quintile	226 (17.0)	135 (15.1)	169 (15.4)	10723 (15.7)	10722 (15.8)
3rd Quintile	247 (18.6)	146 (16.4)	222 (20.3)	12680 (18.6)	12673 (18.6)
4th Quintile	338 (25.4)	255 (28.6)	262 (23.9)	17605 (25.8)	17578 (25.8)
5th Quintile	347 (26.1)	251 (28.1)	310 (28.3)	18811 (27.6)	18721 (27.5)
**Median person time**, years	9.3	3.9	8.7	7.5	7.5
**Total person-time**, years	13171.95	5961.20	10615.32	618732.62	617014.87

© 2023 Jackson SS et al. *JAMA Network Open.*

**Died,** n (%)	70 (5.3)	13 (1.5)	53 (4.8)	1951 (2.9)	1608 (2.4)
**Cause of death,**[c] n (%)					
Certain infectious and parasitic diseases	≤5 (-)	0 (0)	0 (0)	19 (1.0)	29 (1.9)
Codes for special purposes	≤5 (-)	0 (0)	0 (0)	12 (0.7)	≤5 (-)
Congenital malformations, deformations, and chromosomal abnormalities	0 (0)	0 (0)	0 (0)	7 (0.4)	≤5 (-)
Diseases of the blood and blood-forming organs and certain disorders involving the immune mechanism	0 (0)	0 (0)	0 (0)	6 (0.3)	6 (0.4)
Diseases of the circulatory system	16 (25.0)	≤5 (-)	14 (26.4)	506 (27.5)	294 (19.3)
Diseases of the digestive system	≤5 (-)	≤5 (-)	7 (13.2)	152 (8.3)	111 (7.3)
Diseases of the eye and adnexa	0 (0)	0 (0)	0 (0)	0 (0)	≤5 (-)
Diseases of the genitourinary system	0 (0)	0 (0)	0 (0)	18 (1.0)	17 (1.1)
Diseases of the musculoskeletal system and connective tissue	0 (0)	0 (0)	0 (0)	7 (0.4)	17 (1.1)
Diseases of the nervous system	≤5 (-)	0 (0)	≤5 (-)	84 (4.6)	86 (5.7)
Diseases of the respiratory system	8 (12.5)	≤5 (-)	≤5 (-)	203 (11.0)	156 (10.3)
Diseases of the skin and subcutaneous tissue	0 (0)	0 (0)	0 (0)	6 (0.3)	≤5 (-)
Endocrine, nutritional, and metabolic diseases	≤5 (-)	≤5 (-)	≤5 (-)	35 (1.9)	19 (1.3)
External causes of morbidity and mortality	8 (12.5)	≤5 (-)	10 (18.9)	169 (9.2)	98 (6.4)
Mental and behavioral disorders	≤5 (-)	≤5 (-)	0 (0)	67 (3.6)	83 (5.5)
Neoplasms	13 (20.3)	≤5 (-)	12 (22.6)	530 (28.8)	580 (38.2)
Pregnancy, childbirth, and the puerperium	0 (0)	0 (0)	0 (0)	0 (0)	≤5 (-)
Symptoms, signs and abnormal clinical and laboratory findings, not elsewhere classified	≤5 (-)	≤5 (-)	≤5 (-)	19 (1.0)	9 (0.6)

CPRD = Clinical Practice Research Datalink; n = number; SE = standard error; TGD = transgender and gender diverse.

Sex assigned at birth for transgender individuals were it was previously unknown was imputed using multiple imputation with chained equations. Body mass index, smoking status, and alcohol use were imputed for the entire cohort.

[a] Asian was defined as Bangladeshi, Chinese, Indian, Pakistani, or Other Asian; Black was defined as Black African, Black Caribbean, or Black Other; and Other defined as Mixed or Other.

[b] Practice Level Index of Multiple Deprivation

[c] CPRD requires suppression of counts ≤5. As such, no proportions are reported for these cells.

© 2023 Jackson SS et al. *JAMA Network Open.*

**eTable 10**. Overall and Cause-Specific Mortality Rate Ratios for Transgender and Gender Diverse Individuals Compared to Cisgender Individuals in the United Kingdom's Clinical Practice Research Datalink (Without Imputation)									
	**Transfeminine individuals**			**Transmasculine individuals**			**TGD individuals unknown sex assigned at birth**		
**Cause of death**	**Number who died (No)[a]**	**MRR (95% CI)** Compared to cis men	**MRR (95% CI)** Compared to cis women	**Number who died (No)[a]**	**MRR (95% CI)** Compared to cis men	**MRR (95% CI)** Compared to cis women	**Number who died (No)[a]**	**MRR (95% CI)** Compared to cis men	**MRR (95% CI)** Compared to cis women
Overall	70	1.19 (0.94–1.51)	1.54 (1.21–1.89)	13	1.11 (0.65–1.91)	1.43 (0.82–2.49)	53	1.71 (1.31–2.23)	2.11 (1.61–2.78)
Certain infectious and parasitic diseases	≤5	1.82 (0.41–8.01)	1.02 (0.24–4.43)	0	-	-	0	-	-
Diseases of the circulatory system	16	0.85 (0.51–1.43)	1.11 (0.64–1.93)	≤5	0.64 (0.16–2.59)	1.14 (0.35–3.70)	14	1.17 (0.72–1.89)	1.57 (0.88–2.81)
Diseases of the digestive system	≤5	1.03 (0.43–2.46)	0.97 (0.39–2.39)	≤5	1.08 (0.17–6.91)	1.01 (0.16–6.28)	7	1.68 (0.83–3.40)	1.66 (0.80–3.44)
Diseases of the nervous system	≤5	0.73 (0.19–2.88)	0.54 (0.15–1.98)	0	-	-	≤5	1.04 (0.28–3.79)	0.71 (0.19–2.69)
Diseases of the respiratory system	8	1.06 (0.52–2.17)	1.07 (0.53–2.16)	≤5	0.76 (0.13–4.47)	1.09 (0.20–6.07)	8	0.83 (0.32–2.13)	0.77 (0.31–1.95)
Endocrine, nutritional and metabolic diseases	≤5	0.69 (0.07–6.90)	1.21 (0.16–9.31)	≤5	3.58 (0.85–15.14)	3.41 (0.26–43.64)	≤5	1.97 (0.62–6.25)	3.47 (0.99–11.86)
External causes of mortality	8	1.49 (0.70–3.15)	1.52 (0.75–3.06)	≤5	1.30 (0.44–3.84)	1.28 (0.43–3.76)	10	1.82 (1.00–3.33)	2.03 (1.11–3.70)
Mental and behavioral disorders	≤5	1.53 (0.47–5.00)	1.17 (0.43–3.19)	≤5	2.28 (0.63–8.30)	1.48 (0.26–8.43)	0	-	-
Neoplasms	13	0.72 (0.42–1.24)	0.48 (0.28–0.83)	≤5	0.99 (0.33–2.99)	0.69 (0.23–2.17)	12	1.11 (0.69–1.78)	0.71 (0.43–1.17)
Symptoms, signs and abnormal clinical and laboratory findings, not elsewhere classified	≤5	7.57 (2.42–23.67)	10.02 (2.97–39.64)	≤5	8.60 (0.88–83.80)	14.02 (1.56-126.25)	≤5	1.65 (0.20–13.94)	2.74 (0.24–31.02)

95% CI = 95% confidence interval; MRR = mortality rate ratio; No = number; TGD = transgender and gender diverse.
Models were estimated using Poisson regression adjusted for index age, index year, race/ethnicity (White, Black, and Asian or another or unknown race/ethnicity), index of multiple deprivation (quintiles), smoking status (current, former, never, or missing), alcohol use (current, former, never, or missing), body mass index (underweight or healthy weight, overweight, obese, or missing) and practice.
[a] CPRD requires suppression of counts ≤5.

© 2023 Jackson SS et al. *JAMA Network Open.*

eTable 11. Overall and Cause-Specific Mortality Rate Ratios for Transgender and Gender Diverse Individuals Compared to Cisgender Individuals in the United Kingdom's Clinical Practice Research Datalink (Only Patients Who Could Have Died During or After 1998)

	**Transfeminine individuals**			**Transmasculine individuals**		
**Cause of death**	**Number who died (n)** [a]	**MRR (95% CI)** Compared to cisgender men	**MRR (95% CI)** Compared to cisgender women	**Number who died (n)** [a]	**MRR (95% CI)** Compared to cisgender men	**MRR (95% CI)** Compared to cisgender women
Overall	101	1.39 (1.13–1.70)	1.67 (1.36–2.05)	29	1.30 (0.88–1.92)	1.59 (1.07–2.36)
Certain infectious and parasitic diseases	≤5	1.83 (0.42–8.00)	0.92 (0.22–3.92)	0	-	-
Diseases of the circulatory system	25	1.06 (0.71–1.58)	1.53 (1.02–2.29)	≤5	0.72 (0.28–1.89)	1.11 (0.41–2.96)
Diseases of the digestive system	9	1.34 (0.70–2.57)	1.33 (0.68–2.56)	≤5	1.20 (0.27–5.45)	1.20 (0.26–5.56)
Diseases of the nervous system	≤5	0.65 (0.18–2.27)	0.48 (0.14–1.70)	≤5	1.17 (0.16–8.48)	0.75 (0.10–5.49)
Diseases of the respiratory system	9	0.87 (0.45–1.68)	0.87 (0.45–1.70)	≤5	1.28 (0.43–3.82)	1.37 (0.46–4.09)
Endocrine, nutritional and metabolic diseases	≤5	1.50 (0.40–5.68)	2.54 (0.64–10.09)	≤5	2.36 (0.32–17.34)	3.55 (0.50–24.96)
External causes of mortality	13	1.52 (0.88–2.62)	1.77 (1.00–3.11)	≤5	1.26 (0.50–3.22)	1.43 (0.55–3.73)
Mental and behavioral disorders	≤5	0.97 (0.34–2.73)	0.89 (0.32–2.45)	≤5	1.05 (0.14–8.07)	0.69 (0.09–5.11)
Neoplasms	20	0.78 (0.50-1.21)	0.51 (0.33-0.80)	7	1.13 (0.53-2.45)	0.77 (0.35-1.67)
Symptoms, signs and abnormal clinical and laboratory findings, not elsewhere classified*	≤5	4.66 (1.65–13.16)	4.02 (0.49–32.80)	≤5	8.93 (2.81–28.34)	8.76 (1.05–69.21)

95% CI = 95% confidence interval; MRR = mortality rate ratio; and n = number.
Models were estimated using Poisson regression adjusted for index age, index year, index of multiple deprivation (continuous), body mass index (underweight or healthy weight, overweight, or obese) and practice.
Missing sex assigned at birth was imputed using multiple imputation. Models were estimated using Poisson regression adjusted for index age, index year, race/ethnicity (White, Black, and Asian or another or unknown race/ethnicity), index of multiple deprivation (quintiles), smoking status (current, former, or never), alcohol use (current, former, or never), body mass index (underweight or healthy weight, overweight, or obese) and practice.
[a] CPRD requires suppression of counts ≤5.

© 2023 Jackson SS et al. *JAMA Network Open.*

eTable 12. Mortality Rate Ratios for Deaths Due to Select Causes of Death Among Transgender and Gender Diverse Individuals Compared to Cisgender Individuals in the United Kingdom's Clinical Practice Research Datalink (Without Imputation)

	**All transgender and gender diverse individuals**		
**Cause of death**	**Number who died (n)** [a]	**MRR (95% CI)** Compared to cisgender men	**MRR (95% CI)** Compared to cisgender women
**External causes of mortality**			
Suicide/homicide	9	3.01 (1.45–6.22)	5.65 (2.66–11.99)
Accidental poisoning	6	2.43 (1.12–5.28)	5.29 (2.37–11.83)
**Neoplasms**			
Gastrointestinal	14	1.55 (0.81–2.95)	2.25 (1.16–4.35)
Lung	9	1.18 (0.60–2.35)	1.22 (0.63–2.39)
**Endocrine, nutritional, and metabolic diseases**	≤5	2.19 (0.90–5.34)	3.21 (1.22–8.41)
**Symptoms, signs and abnormal clinical and laboratory findings, not elsewhere classified**			
Other ill-defined and unspecified causes of mortality	≤5	5.67 (2.04–15.79)	19.54 (5.64–66.74)

95% CI = 95% confidence interval; MRR = mortality rate ratio; and n = number.
Models were estimated using Poisson regression adjusted for index age, index year, race/ethnicity (White, Black, and Asian or another or unknown race/ethnicity), index of multiple deprivation (quintiles), smoking status (current, former, or never), alcohol use (current, former, never), body mass index (underweight or healthy weight, overweight, obese) and practice.
[a] CPRD requires suppression of counts ≤5.

eTable 13. Mortality Rate Ratios for Deaths Due to Select Causes Among Transgender and Gender Diverse Individuals Compared to Cisgender Individuals in the United Kingdom's Clinical Practice Research Datalink (Only Patients Who Could Have Died During or After 1998)

	**All transgender and gender diverse individuals**		
**Cause of death**	**Number who died (n)***	**MRR (95% CI)** Compared to cisgender men	**MRR (95% CI)** Compared to cisgender women
**External causes of mortality**			
Suicide/homicide	9	3.06 (1.51–6.20)	5.61 (2.65–11.86)
Accidental poisoning	6	1.97 (0.85–4.57)	4.48 (1.82–11.05)
**Neoplasms**			
Gastrointestinal	14	1.60 (0.92–2.76)	2.27 (1.30–3.96)
Lung	9	1.26 (0.92–2.76)	1.20 (0.61–2.37)
**Endocrine, nutritional, and metabolic diseases**	≤5	1.80 (0.68–4.72)	2.95 (1.08–8.06)
**Symptoms, signs and abnormal clinical and laboratory findings, not elsewhere classified**			
Other ill-defined and unspecified causes of mortality	≤5	5.38 (2.00–14.50)	19.63 (5.50–66.71)

95% CI = 95% confidence interval; MRR = mortality rate ratio; and n = number.
Missing covariate data were imputed using multiple imputation. Models were estimated using Poisson regression adjusted for continuous index age, continuous index year, race/ethnicity (White, Black, and Asian or another or unknown race/ethnicity), index of multiple deprivation (quintiles), smoking status (current, former, never), alcohol use (current, former, never), body mass index (underweight or healthy weight, overweight, obese) and practice.
[a] CPRD requires suppression of counts ≤5.

© 2023 Jackson SS et al. *JAMA Network Open.*

eFigure. Flow Chart of Final Analysis Cohort Combining CPRD GOLD and CPRD Aurum

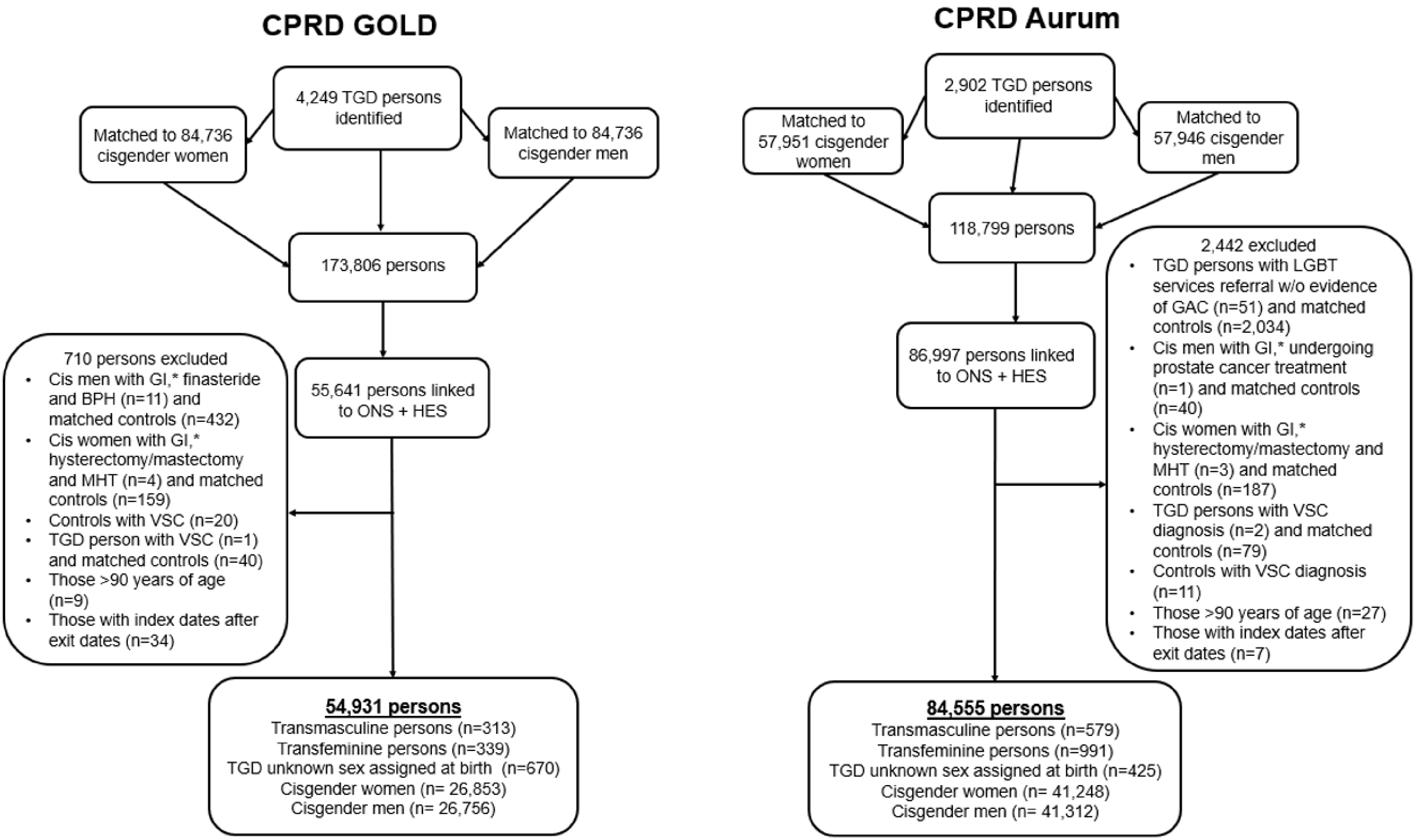

*Individuals with gender incongruence codes in the medical record who were later identified to be cisgender people with sex-specific medical conditions that required sex steroid hormone therapy or surgery.

BPH = benign prostate hypertrophy; cis = cisgender; GAC = gender-affirming care; GI = gender incongruence; HES = Hospital and Episodes Statistics; LGBT = lesbian, gay, bisexual, and transgender; MHT = menopausal hormone therapy; ONS = Office of National Statistics; and VSC = variations of sex characteristics

© 2023 Jackson SS et al. *JAMA Network Open.*
