# Supplementary material for: Analysis of Mortality Among Transgender and Gender Diverse Adults in England
Source: JAMA Netw Open. 2023 Jan 30;6(1):e2253687. doi: 10.1001/jamanetworkopen.2022.53687 (PMC9887492; doi:10.1001/jamanetworkopen.2022.53687)
Supplement: Supplement 2. — Data Sharing Statement [file jamanetwopen-e2253687-s002.pdf]

Data Sharing Statement

Jackson. Analysis of Mortality Among Transgender and Gender Diverse Adults in England. *JAMA Netw Open*. Published January 30, 2023. doi:10.1001/jamanetworkopen.2022.53687

Data
Data available: No
